# Astrocyte‐Derived Extracellular Vesicular miR‐143‐3p Dampens Autophagic Degradation of Endothelial Adhesion Molecules and Promotes Neutrophil Transendothelial Migration after Acute Brain Injury

**DOI:** 10.1002/advs.202305339

**Published:** 2023-12-03

**Authors:** Xun Wu, Haixiao Liu, Qing Hu, Jin Wang, Shenghao Zhang, Wenxing Cui, Yingwu Shi, Hao Bai, Jinpeng Zhou, Liying Han, Leiyang Li, Yang Wu, Jianing Luo, Tinghao Wang, Chengxuan Guo, Qiang Wang, Shunnan Ge, Yan Qu

**Affiliations:** ^1^ Department of Neurosurgery Tangdu Hospital the Fourth Military Medical University Xi'an Shaanxi 710038 China; ^2^ Department of Neurosurgery The Second Hospital of Hebei Medical University Shijiazhuang Hebei 050000 China; ^3^ Department of Neurosurgery West Theater General Hospital Chengdu Sichuan 610083 China

**Keywords:** acute brain injury, cell adhesion molecules, endothelial cells, extracellular vesicles, neutrophils

## Abstract

Pivotal roles of extracellular vesicles (EVs) in the pathogenesis of central nervous system (CNS) disorders including acute brain injury are increasingly acknowledged. Through the analysis of EVs packaged miRNAs in plasma samples from patients with intracerebral hemorrhage (ICH), it is discovered that the level of EVs packaged miR‐143‐3p (EVs‐miR‐143‐3p) correlates closely with perihematomal edema and neurological outcomes. Further study reveals that, upon ICH, EVs‐miR‐143‐3p is robustly secreted by astrocytes and can shuttle into brain microvascular endothelial cells (BMECs). Heightened levels of miR‐143‐3p in BMECs induce the up‐regulated expression of cell adhesion molecules (CAMs) that bind to circulating neutrophils and facilitate their transendothelial cell migration (TEM) into brain. Mechanism‐wise, miR‐143‐3p directly targets ATP6V1A, resulting in impaired lysosomal hydrolysis ability and reduced autophagic degradation of CAMs. Importantly, a VCAM‐1–targeting EVs system to selectively deliver miR‐143‐3p inhibitor to pathological BMECs is created, which shows satisfactory therapeutic effects in both ICH and traumatic brain injury (TBI) mouse models. In conclusion, the study highlights the causal role of EVs‐miR‐143‐3p in BMECs’ dysfunction in acute brain injury and demonstrates a proof of concept that engineered EVs can be devised as a potentially applicable nucleotide drug delivery system for the treatment of CNS disorders.

## Graphical abstract

Upon acute brain injury, activated astrocytes transfer miR‐143‐3p into brain microvascular endothelial cells (BMECs) through EVs, which then block the autophagic degradation of several key cell adhesion molecules (CAMs) by targeting ATP6V1A, and promote excessive neutrophil transendothelial cell migration (TEM). Engineered EVs that display VCAM‐1‐targeting peptide on the surface selectively deliver miR‐143‐3p inhibitor to pathological BMECs and prevent secondary brain injury.

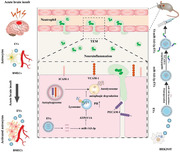

## 1 Introduction

The blood‐brain barrier (BBB) is a highly specialized barrier that separates the peripheral circulation from the central nervous system (CNS).^[^ ^1^, ^2^ ^]^ Brain microvascular endothelial cells (BMECs) form a monolayer cell structure that constitutes the major component of BBB. In a physiological state, BMECs act as a gatekeeper, blocking the entry of toxins, pathogens, and peripheral circulating cells into brain.^[^ ^3^, ^4^ ^]^ However, early after the onset of an acute brain injury like intracerebral hemorrhage (ICH) and traumatic brain injury (TBI), BMECs typically undergo activation and exhibit heightened expression of inflammatory cell adhesion molecules (CAMs) including ICAM‐1, VCAM‐1, PECAM‐1 and E‐selectin.^[^ ^5^, ^6^, ^7^ ^]^ CAMs play a pivotal role in the adhesion of circulating immune cells to the inflamed BMECs, thereby facilitating their transmigration across the endothelial barrier into the brain.^[^ ^8^, ^9^, ^10^ ^]^ Peripheral immune cell infiltration has been linked to secondary brain injury, and principally inflicts neuronal death through the release of proinflammatory cytokines, reactive oxygen species, matrix metalloproteinases (MMPs), and proteases.^[^ ^11^, ^12^, ^13^ ^]^ Hence, defining the molecular basis of CAMs’ expression and blocking transendothelial migration (TEM) of circulating immune cells are of vital importance for attenuating secondary brain injury.

Extracellular vesicles (EVs) are lipid bilayer structures that carry various bioactive molecules.^[^ ^14^, ^15^ ^]^ EVs are critical for intercellular communication by transferring their cargo to the recipient cell to elicit functional responses.^[^ ^16^, ^17^ ^]^ In addition to their involvement in various physiological processes, EVs play an important pathophysiological role in multiple human diseases including cancer progression, neurological diseases, cardiovascular dysfunction, metabolic disorders, and so on.^[^ ^18^, ^19^, ^20^ ^]^ MicroRNAs (miRNAs) are identified as small non‐coding RNAs that regulate gene expression in cells through translational regulation and are involved in nearly all biological processes.^[^ ^21^, ^22^ ^]^ EVs packaged miRNAs (EVs‐miRNAs) have been implicated in the pathogenesis of CNS diseases and act as a promising target for treatment.^[^ ^23^, ^24^ ^]^ In the study, we aimed to explore the potential role of EVs‐miRNAs in the pathological progress after acute brain injury.

Once the causal role of EVs‐miRNAs in pathological progress following acute brain injury is determined, it becomes imperative to explore nucleotide‐based therapies for potential medical practice in the future. However, systemic administration of nucleotides targeting dysregulated miRNAs has been associated with undesirable pharmacokinetic characteristics. These include rapid degradation within the body and limited cellular uptake, resulting in low bioavailability specifically in target cells, and unintended side effects in non‐target tissues^[^ ^.25^ ^]^ Consequently, the development of a drug delivery system that can safeguard content from degradation and target the lesion site is increasingly garnering the attention of scholars.^[^ ^26^, ^27^, ^28^ ^]^ It is noteworthy that EVs, in addition to their function as intercellular messengers, possess immense potential as gene‐delivery vehicles due to their promising biocompatibility and biodegradability, low toxicity, and remarkable capacity to safeguard endogenous biologically active constituents.^[^ ^29^, ^30^ ^]^ Furthermore, EVs can be engineered to display ligands of target molecules, thereby facilitating cell‐targeted delivery of therapeutic nucleotides.^[^ ^31^, ^32^, ^33^ ^]^ Hence, engineered EVs hold promise as a potentially applicable nucleotide drug delivery system for targeting lesion tissues.

In the current investigation, a miRNA sequencing process was conducted, and the level of EVs packaged miR‐143‐3p (EVs‐miR‐143‐3p) was discovered to be closely associated with neurological outcomes in patients with ICH. Subsequently, we identified that upon acute injury, activated astrocytes robustly secreted EVs‐miR‐143‐3p that was then shuttled into BMECs. Then the causal role of EVs‐miR‐143 in BMECs’ barrier dysfunction was determined, and investigations of the molecular mechanisms were carried out. Lastly, we constructed a novel VCAM‐1–targeting, miR‐143‐3p inhibitor encapsulated EVs to experimentally characterize the therapeutic benefits of specifically targeting miR‐143‐3p in pathological BMECs using both ICH and TBI mouse models.

## 2 Result

### 2.1 Peripheral Level of EVs‐miR‐143‐3p Correlated With Perihematomal Edema, As Well As Neurological Outcomes, In Patients With ICH

**Figure 1:**
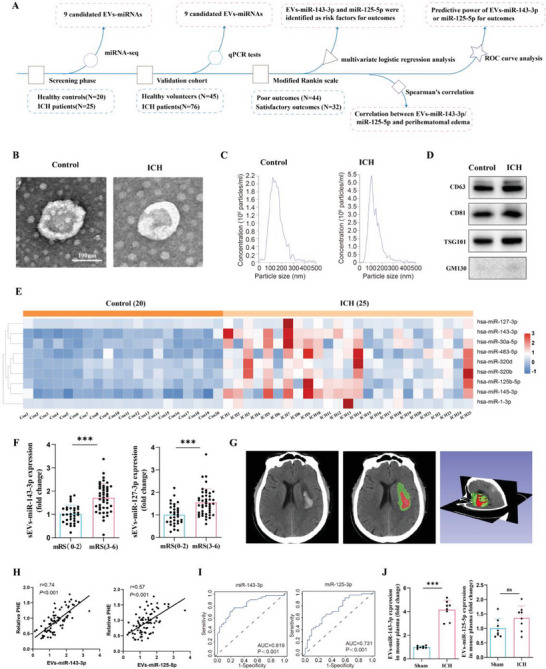
Serum level of EVs‐miR‐143‐3p correlated with perihematomal edema, as well as neurological outcomes in patients with ICH. A) Scheme showing the design of the clinical research section. B) Transmission electron microscopy (TEM) analysis and C) nanoparticle tracking analysis (NTA) of the isolated EVs. Scale bar, 100 nm. D) Western blotting analysis of the EVs marker proteins including CD63, CD81, and TSG101, and cis‐Golgi marker protein, GM130. E) Heatmap showing the differently expressed EVs‐packaged miRNAs between ICH patients and healthy controls in miRNA‐seq data (ICH = 25, CON = 20; log^2^FC ≥2 and *p <* 0.05). F) Relative plasma EVs‐miR‐143‐3p and EVs‐miR‐125‐5p levels on the 1st day in patients with good (mRS 0 to 2) outcomes versus poor (mRS 3 to 6) outcomes 6 months later. *n =* 32 in good outcome group and *n =* 44 in poor outcome group. mRS: modified Rankin score. G) The representative CT image and 3D reconstruction process denote a hematoma and a perihematomal region in the right hemisphere of the brain from patients with ICH, where the red color represents the hematoma area and the green color represents the edema area. H) Pearson correlation coefficient (*r*) and *P* value (*P*) between plasma EVs‐miR‐143‐3p or EVs‐miR‐125‐5p and relative PHE (PHE/hematoma) in ICH patients (*n =* 76). PHE: perihematomal edema. I) ROC curve for individual plasma EVs‐miR‐143‐3p or EVs‐miR‐125‐5p expression on day 1 to separate good (mRS = 0–2) outcomes from poor (mRS = 3–6) outcomes 6 months later. J) Detection of EVs‐miR‐143‐3p and EVs‐miR‐125‐5p levels in the plasma of mice (*n =* 8 per group; Student's t‐test). Data are expressed as mean ± SD. *p < 0.05; **p < 0.01; ***p < 0.001.

Given the difficulty of acquiring human brain tissues for EVs detection, peripheral serum was chosen as a surrogate subject, which could be obtained easily and partly reflect the molecular alterations in brain.^[^ ^34^, ^35^, ^36^ ^]^ EVs were isolated from plasma by ultracentrifugation. Transmission electron microscopy (TEM), nanoparticle tracking analysis (NTA), and western blotting (WB) analysis were performed to validate our EVs preparations (**Figure** **1**B–D). During the preliminary screening, miRNA‐seq was performed to determine expression profiles of EVs‐packaged miRNAs in patients with ICH (*n =* 25) and age‐matched healthy controls (*n =* 20). Setting log^2^FC ≥2, TPM>10 as the threshold to define differentially regulated EVs‐miRNAs, nine miRNAs including hsa‐miR‐1‐3p, hsa‐miR‐127‐3p, hsa‐miR‐483‐5p, hsa‐miR‐320d, hsa‐miR‐143‐3p, hsa‐miR‐125b‐5p, hsa‐miR‐145‐3p, hsa‐miR‐30a‐5p and hsa‐miR‐320b were found to be differentially expressed in patients with ICH and healthy controls (Figure 1E). Based on qPCR results in validation and replication experiments, the altered patterns of all nine miRNAs were consistent with miRNA‐seq results (Figure S1A, Supporting Information).

Next, we evaluated whether alterations of the nine EV‐miRNAs were associated with disease progression and neurological outcomes in ICH patients. Multivariate logistic regression analysis was performed to determine the risk factors for ICH patients’ neurological outcomes at 6 months after disease onset measured by a modified Rankin scale (mRS). Among the nine candidate EV‐miRNAs, EVs‐hsa‐miR‐143‐3p and hsa‐miR‐125‐5p were found to be the risk factors for patients’ neurological outcomes, and differed between patients who eventually achieved satisfactory outcomes and those with poor outcomes; but this finding was not observed for the other seven miRNAs (**Table** **1**; Figure 1F). The volume of each patient's perihematomal edema (PHE), which is a hallmark of secondary brain injury, was quantified using 3D‐slicer software (Figure 1G). Pearson's correlation coefficient (r2) indicated a strong correlation between plasma levels of EVs‐miR‐143‐3p and PHE after ICH (Figure 1H). Furthermore, receiver operating characteristic (ROC) curve analysis was performed to determine the predictive power of EVs‐hsa‐miR‐143‐3p and hsa‐miR‐125‐5p levels for ICH outcomes. The area under the curve of EVs‐hsa‐miR‐143‐3p and hsa‐miR‐125‐5p was 0.819 and 0.731, respectively (Figure 1I). EVs‐miR‐143‐3p showed a greater ability to predict ICH neurological outcomes.

**Table 1:** Characteristics of patients with intracerebral hemorrhage (ICH) and multivariate logistic regression analysis.

Characteristic	Satisfactory outcome [*n =* 32]	Unsatisfactory outcome [*n =* 44]	*p*‐value	odds ratio (OR) [95%CI]	*p*‐value
Age (Mean ± SD)	55.13 ± 12.47	63.93 ± 13.23	0.004	1.1(1.011,1.196)	0.027
Gender			0.488		
Female	12(37.5%)	20(45.5%)			
Male	20(62.5%)	24(54.5%)			
Glasgow Coma Scale (GCS)			<0.001	43.206(4.131451.931)	0.002
≥13	22(68.8%)	11(25%)			
<13	10(31.3%)	33(75%)			
pulse (IQ range)	76(68,81)	73(67,79.75)	0.609		
respiratory rate (IQ range)	20(18,20)	18(18,20)	0.079		
white blood cell (WBC) IQ range)	8.415(6.31,10.695)	8.41(6.6825,11.6525)	0.736		
Alanine Aminotransferase (ALT) (IQ range)	22(17.25,34)	23(17.25,32.75)	0.805		
Aspartate Transaminase (AST) (IQ range)	26.5(22.25,34)	29.5(25,37.25)	0.078		
creatinine (Crea) (IQ range)	64.1(51.5,74.675)	60.7(50.475,70.075)	0.449		
urea nitrogen (BUN) (IQ range)	4.535(3.56,6.058)	5.575(4.2775,6.6475)	0.031	1.126(0.927,1.368)	0.23
Estimated Glomerular Filtration Rate (Egfr) (IQ range)	104.25(92.325111.3775)	96.425(81.785105.765)	0.141		
Activated Partial Thromboplastin time (APTT) (IQ range)	24.2(21.7,26.7)	25.9(22.125,28.4)	0.173		
Fibrinogen degradation products (FDP) (IQ range)	2.29(1.9,3.895)	3.47(2.4,11.275)	0.003	1.176(0.964,1.435)	0.109
D‐Dmier (D‐D) (IQ range)	0.67(0.51,0.98)	0.98(0.695,2.1525)	0.010	0.559(0.278,1.122)	0.102
miRNA‐143‐3p (Mean ± SD)	1.06 ± 0.4254	1.715 ± 0.61426	<0.001	20.19(2.161188.641)	0.008
miRNA‐125‐5p (Mean ± SD)	1.0734 ± 0.48176	1.5557 ± 0.59711	<0.001	11.445(1.614,81.140)	0.015
miRNA‐483 (Mean ± SD)	1.0772 ± 0.56741	0.9075 ± 0.62711	0.23		
miRNA‐320b (Mean ± SD)	1.0381 ± 0.53113	1.2005 ± 0.44740	0.153		
miRNA‐145‐3p (Mean ± SD)	1.0159 ± 0.46494	1.0843 ± 0.43050	0.511		
miRNA‐1‐3p (Mean ± SD)	1.0575 ± 0.56653	1.0207 ± 0.45837	0.755		
miRNA‐320d (Mean ± SD)	1.02 ± 0.43731	1.097 ± 0.4516	0.459		
miRNA‐30a‐5p (Mean ± SD)	1.0659 ± 0.38449	1.1214 ± 0.39067	0.457		
miRNA‐127b‐3p (Mean ± SD)	1.0513 ± 0.38513	1.1 ± 0.48326	0.638		
Hematoma volumes (mL) (Mean ± SD)	18.45 ± 4.48539	23.9818 ± 5.48704	<0.001	1.306(1.064,1.603)	0.011
hypertension			0.003	0.766(0.101,5.826)	0.797
no	17(53.1%)	9(20.5%)			
yes	15(46.9%)	35(79.5%)			

Subsequently, to determine the causal role of EVs‐hsa‐miR‐143‐3p or hsa‐miR‐125‐5p in ICH, we conducted further fundamental studies. First, we constructed an ICH mouse model. Consistent with the phenomenon observed in clinical patients, we discovered a significant elevation in the plasma level of EVs‐miR‐143‐3p in ICH mice. However, we didn't observe significant changes in the plasma level of EVs‐miR‐125‐5p (Figure 1J). Accordingly, miR‐143‐3p was selected as the candidate miRNA for further investigation in animal studies.

### 2.2 EVs‐miR‐143‐3p was Mainly Secreted By Activated Astrocytes After ICH and Could Shuttle Into BMECs

**Figure 2:**
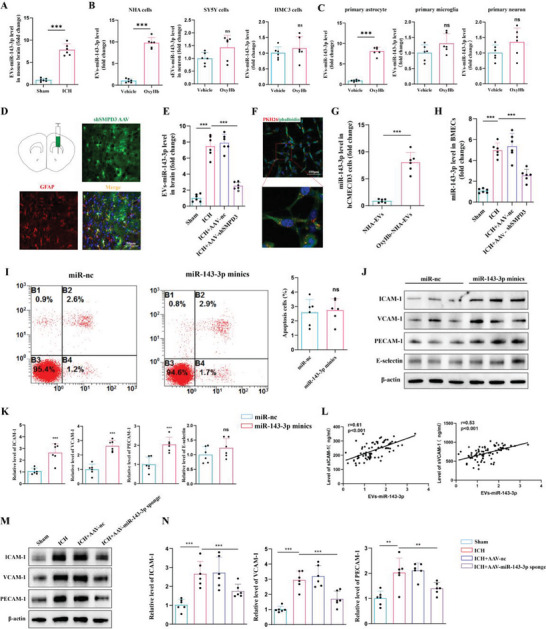
Activated astrocytes transferred miR‐143‐3p into BMECs through EVs and promoted endothelial CAMs expression after ICH. A) qRT‐PCR analysis of the level of EVs‐miR‐143‐3p in the perihematomal brain tissues of mice at 24 h post‐ICH, compared to that in sham group mice (*n =* 6 per group; Student's t‐test). B) The level of miR‐143‐3p in EVs derived from the culture supernatants of human SH‐SY5Y (neuron), NHA (astrocyte), or HMC3 (microglia) cell lines with or without OxyHb treatment (*n =* 6 per group, Student's t‐test). C) The level of miR‐143‐3p in EVs derived from the culture supernatants of primary neurons, astrocytes, and microglia with or without OxyHb treatment (*n =* 6 per group, Student's t‐test). D) Representative immunofluorescence images of mouse brain sections after stereotactically injecting AAV‐SMPD3‐shRNA that contains a GFAP‐specific promoter for 3 weeks. Scale bars, 50 µm. E) qRT‐PCR analysis of the level of EVs‐miR‐143‐3p in the perihematomal brain tissues of ICH mice with or without astrocytes‐specific knockdown of SMPD3 (*n =* 6 per group, Student's t‐test). F) Confocal imaging showing the absorption of PKH26‐labeled EVs that derived from NHAs cells in hCMEC/D3 cells. hCMEC/D3, human brain microvascular endothelial cell line. Scale bar: 100 µm. G) The level of miR‐143‐3p in hCMEC/D3 cells was quantified after the incubation of EVs derived from NHAs (*n =* 6 per group, Student's t‐test). H) qRT‐PCR analysis of the level of miR‐143‐3p in isolated BMECs from ICH mice with or without astrocytes‐specific knockdown of SMPD3 (*n =* 6 per group, one‐way ANOVA). I) Cell apoptosis was analyzed through annexin V‐FITC flow cytometry in hCMEC/D3 cells transfected with miR‐143‐3p minics or miR‐nc (*n =* 6 per group, Student's t‐test). J,K) Western blot assay of the expression of CAMs including ICAM‐1, VCAM‐1, PECAM‐1, and E‐selectin in hCMEC/D3 cells transfected with miR‐143‐3p minics or miR‐nc (*n =* 6 per group, Student's t‐test). L) Pearson analyses of the correlation between miR‐143‐3p and the serum levels of s‐VCAM‐1, and s‐ICAM‐1 within 24 h after ICH onset in patients (*n =* 76). s‐VCAM‐1: soluble VCAM‐1; s‐ICAM‐1: soluble ICAM‐1. (M‐N) Western blot assay of PECAM‐1, VCAM‐1, and ICAM‐1 in BMECs isolated from the perihematomal brain tissues of ICH mice with or without stereotactically injecting astrocytes‐specific AAV‐ miR‐143‐3p sponge. (*n =* 6 per group, Student's t‐test). Data are expressed as mean ± SD. *p < 0.05; **p < 0.01; ***p < 0.001.

It is noteworthy that miR‐143‐3p is a brain‐specific miRNA.^[^ ^37^, ^38^, ^39^ ^]^ Therefore, it is plausible to assume that the increased level of EVs‐miR‐143‐3p in plasma of ICH patients may largely reflect the altered level of EVs‐miR‐143‐3p in brain. As a matter of fact, we observed a significant increase in the EVs‐miR‐143‐3p level in brain tissues after ICH in mice (**Figure** **2**A). Consequently, we proceeded to investigate which type of brain cells are responsible for ICH‐induced elevated levels of EVs‐miR‐143‐3p. Several human cell lines, including SH‐SY5Y (neuron), NHA (astrocyte), and HMC3 (microglia), were cultured respectively to establish an in vitro ICH model through OxyHb treatment. The supernatant was collected and EVs were isolated to detect changes in the level of miR‐143‐3p. The research findings revealed that treatment with OxyHb significantly up‐regulated the level of miR‐143‐3p in EVs isolated from the supernatant of astrocytes, but not from other cell lines (Figure 2B). Similar results were observed in cultured primary mouse neurons, microglia, and astrocytes (Figure 2C). Moreover, we investigated whether inhibiting the release of astrocytic EVs could suppress the increase in EVs‐miR‐143‐3p level in ICH mice. Neutral sphingomyelinase 2 (nSMase2), a product of the SMPD3 gene, is an essential enzyme for the synthesis and release of EVs^[^ ^.40^ ^]^ We specifically knockdown SMPD3 in astrocytes by stereotactically injecting AAV‐SMPD3‐shRNA that contains a GFAP‐specific promoter (Figure 2D). Importantly, we found that after inhibiting the secretion of astrocytic EVs, the level of EVs‐miR‐143‐3p in brain pool was no longer elevated after ICH (Figure 2E). These results suggested that the increased EVs‐miR‐143‐3p in ICH is mainly secreted by activated astrocytes.

Astrocytes and endothelial cells, which are structurally and functionally interdependent, together form the BBB. We wondered whether astrocytes‐derived EVs‐miR‐143‐3p can be transferred into BMECs and thereby affect endothelial function. First, we cultured human hCMEC/D3 cells and treated them with PKH26‐labeled EVs isolated from astrocytic cells’ supernatant for 24 h. Subsequently, confocal microscopy analysis showed that PKH26‐labeled EVs were presented in hCMEC/D3 cells, indicating that astrocyte‐derived EVs can be internalized by BMECs (Figure 2F).

Meanwhile, we found that the level of miR‐143‐3p was significantly higher in hCMEC/D3 cells treated with EVs derived from activated astrocytic cells (Figure 2G). Moreover, in the in vivo studies, we isolated endothelial cells from mouse brains using CD31 MicroBeads and found that the level of miR‐143‐3p in BMECs was significantly up‐regulated in ICH group mice when compared to the sham group. Inhibiting the release of astrocytic EVs through specific knockdown of SMPD3 in astrocytes largely inhibited the increase of miR‐143‐3p in BMECs after ICH (Figure 2H). Together, these results showed that miR‐143‐3p were enriched in EVs derived from activated astrocytes in ICH and could be delivered into BMECs.

### 2.3 EVs‐miR‐143‐3p Led to the Increased Expressions of CAMs in Recipient BMECs

To explore the potential impact of elevated levels of miR‐143‐3p in BMECs, we first transfected miR‐143‐3p minics into hCMEC/D3 cells. Flow cytometry‐based quantification of early and late apoptotic cells revealed that heightened miR‐143‐3p levels did not directly impact endothelial cell survival (Figure 2I). Then we wondered whether miR‐143‐3p influences the architecture or function of BMECs. The following studies showed that transfection of miR‐143‐3p minics significantly increased the expression of several inflammatory CAMS including ICAM‐1, VCAM‐1, and PECAM‐1 in hCMEC/D3 cells (Figure 2J,K). This finding was also confirmed in murine bEND.3 cell line, mouse brain microvascular endothelial cells (Figure S2A, B Supporting Information). Moreover, we discovered that the level of EVs‐miR‐143‐3p was positively correlated with the plasma levels of soluble ICAM‐1 (sICAM‐1) and soluble VCAM‐1 (sVCAM‐1) in ICH patients, suggesting that this mechanism may also be applied in human subjects (Figure 2L). To obtain more conclusive evidence that astrocytes secreted EVs‐miR‐143‐3p to regulate the expressions of CAMs in BMECs after ICH, we conducted an intervention experiment with the employment of astrocytes‐specific AAV‐miR‐143‐3p sponge. As expected, administration of astrocytes‐specific AAV‐ miR‐143‐3p sponge significantly prevented the up‐regulated expressions of CAMs in BMECs after ICH (Figure 2M,N). Collectively, EVs‐miR‐143‐3p led to the increased cell surface expression of adhesion molecules in recipient BMECs.

### 2.4 miR‐143‐3p Impaired the Autophagic Degradation of CAMs in BMECs

**Figure 3:**
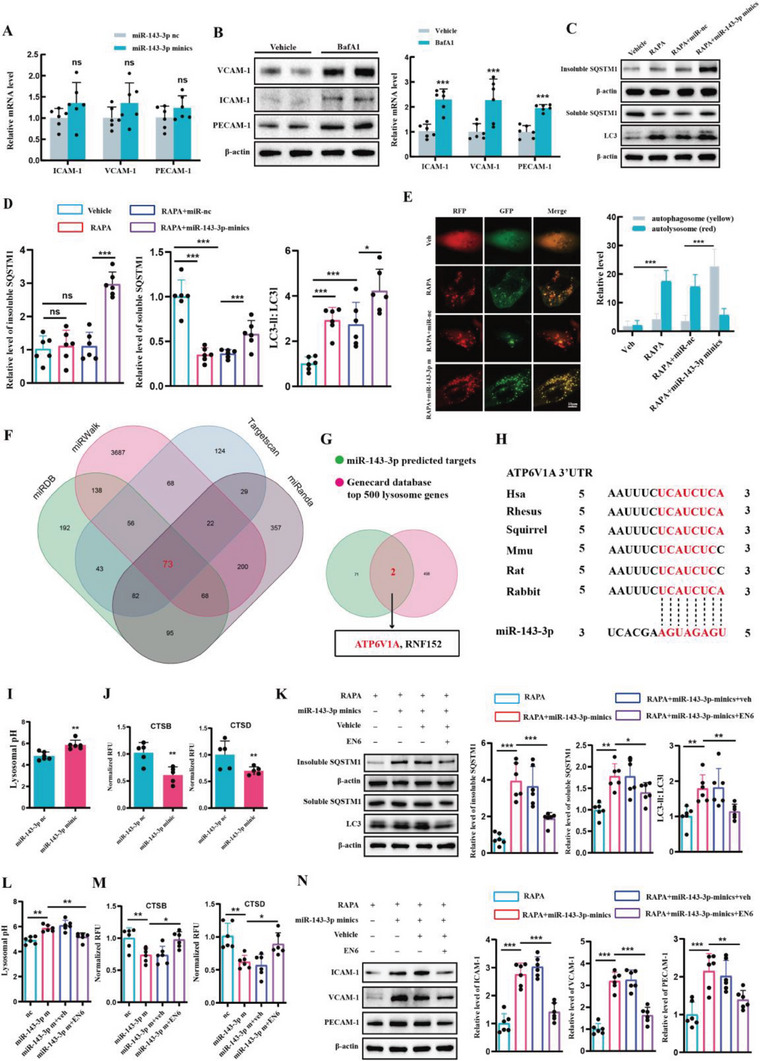
miR‐143‐3p impaired autophagic degradation of CAMs through targeting ATP6V1A. A) PCR assay of the mRNA level of VCAM‐1, ICAM‐1, and PECAM‐1 in hCMEC/D3 cells transfected with miR‐143‐3p minics or miR‐nc (*n =* 6 per group, Student's t‐test). B) WB analysis of the levels of VCAM‐1, ICAM‐1, and PECAM‐1 in human hCMEC/D3 cells after the treatment of the lysosomal proteases inhibitor bafilomycin A1 (*n =* 6 per group, Student's t‐test). C,D) Western blot analysis of the levels of LC3B‐II and SQSTM1 in rapamycin‐treated hCMEC/D3 cells transfected with miR‐143‐3p minics or miR‐nc (*n =* 6 per group, one‐way ANOVA). E) Representative images and quantification of autophagic flux in rapamycin‐treated hCMEC/D3 cells transfected with miR‐143‐3p minics or miR‐NC (*n =* 3 per group, one‐way ANOVA). hCMEC/D3 cells were infected with GFP‐mRFP‐LC3 adenovirus. The intensity of red puncta and yellow puncta were determined. Scale bar: 10 µm. F) Venn diagram showing the targeted genes of miR‐143‐3p predicted by TargetScan, miRanda, miRDB, and miRWalk databases. G) Overlap the predicted genes with the top 500 lysosomal function‐related genes in GeneCards Database. Thus, two candidate genes (ATP6V1A, RNF152) that both were predicted to be the target of miR‐143‐3p and are correlated with lysosomal function were screened out. H) The predicted binding sites of miR‐143‐3p in ATP6V1A‐3′ UTR among different species. I) The detection of Lysosomal pH in hCMEC/D3 cells treated with miR‐nc or miR‐143‐3p minics (*n =* 6 per group, Student's t‐test). J) The measurement of CTSB or CTSD protease activity in hCMEC/D3 cells treated with miR‐nc or miR‐143‐3p minics (*n =* 5 per group, Student's t‐test). K) The effects of EN6 treatment on the protein level of LC3B‐II and SQSTM1 levels in rapamycin‐treated hCMEC/D3 cells transferred with miR‐nc or miR‐143‐3p‐minics (*n =* 6 per group, one‐way ANOVA). EN6: Agonist of ATP6V1A. L) The effects of EN6 treatment on Lysosomal pH in hCMEC/D3 cells treated with miR‐nc or miR‐143‐3p minics (*n =* 6 per group, one‐way ANOVA). M) The effects of EN6 treatment on CTSB or CTSD protease activity in hCMEC/D3 cells treated with miR‐nc or miR‐143‐3p minics (*n =* 6 per group, one‐way ANOVA). N) The effects of EN6 treatment on the protein level of CAMs in hCMEC/D3 cells transferred with miR‐nc or miR‐143‐3p‐minics (*n =* 6 per group, one‐way ANOVA). Data are presented as means ± SD. *p < 0.05, **p < 0.01, ***p < 0.001.

To investigate whether the effects of miR‐143‐3p on the expressions of these CAMs were mediated through direct targeting, we conducted an extensive search for potential miR‐143‐3p binding sites within the 3′‐UTR region of VCAM‐1, ICAM‐1, and PECAM‐1. Our comprehensive bioinformatics analysis, however, did not reveal any direct binding interactions between miR‐143‐3p and these CAMs. Intriguingly, we observed that the overexpression of miR‐143‐3p in BMECs did not lead to any discernible changes in the mRNA expression levels of these molecules (**Figure** **3**A). These findings strongly suggest that differential transcriptional regulation is unlikely to be the primary cause of the observed increase in CAMs’ protein levels. Consequently, we postulate that miR‐143‐3p‐induced up‐regulation of these molecules could potentially arise from dysregulated protein degradation and turnover processes. In eukaryotic cells, proteins or organelles are mainly cleared through two independent but interrelated pathways, namely the ubiquitin‐proteasome pathway and lysosome pathway. To explore the manner of CAMs degradation, the proteasome inhibitor MG132 and the lysosomal protease inhibitor bafilomycin A1 were applied. We found bafilomycin A1 caused PECAM‐1, VCAM‐1, and ICAM‐1 protein to increase in hCMEC/D3 cells (Figure 3B). On the contrary, treatment of cells with MG132 had no effect (Figure S2C,D, Supporting Information). Hence, this suggested that endothelial autophagy may regulate CAMs expression.

Correspondingly, it was observed that miR‐143‐3p indeed impairs autophagic flux in hCMEC/D3 cells. In detail, first, we examined the expression of autophagic substrates, SQSTM1, with or without miR‐143‐3p overexpression (Figure 3C,D). SQSTM1 is subject to degradation via the autophagy‐lysosomal pathway, and its accumulation as aggregates (Triton X‐100 insoluble) in cells occurs when autophagic flux is inhibited.^[^ ^41^, ^42^ ^]^ Treatment with rapamycin, an autophagy agonist, leads to a progressive decrease in the levels of Triton X‐100 soluble SQSTM1, with no significant change observed in the insoluble fractions. Conversely, additional transfection of miR‐143 resulted in a significant increase in the expression of insoluble SQSTM1, suggesting that miR‐143‐3p impedes the digestion of autophagic substrates. To further investigate autophagosome formation in the context of miR‐143‐3p overexpression, we examined the levels of LC3, a marker of autophagosomes. Similarly, hCMEC/D3 cells treated with rapamycin exhibited an elevation in the LC3‐II/LC3‐I ratio, and this ratio was further increased upon miR‐143‐3p overexpression (Figure 3C,D). This finding, in conjunction with the observed changes in SQSTM1, suggests that miR‐143‐3p led to an increase in LC3‐II levels due to impaired lysosomal processing of autophagosomes, rather than an increase in autophagosome generation. To provide more direct evidence regarding impaired lysosomal processing of autophagosomes, cells were transfected with the mRFP‐GFP‐MAP1LC3B‐adenovirus. Green fluorescent protein (GFP) fluorescence is sensitive to acidity and is quenched under acidic conditions, whereas red fluorescent protein (RFP) fluorescence remains stable in the acidic lysosomal environment. Therefore, red puncta (RFP‐only) indicate autolysosomes, whereas yellow puncta (both GFP and RFP fluorescence) represent autophagosomes unfused with lysosomes^[^ ^.43^ ^]^ As indicated in Figure 3E, rapamycin treatment significantly increased RFP‐only puncta, with a relatively low level of yellow puncta. Additionally, miR‐143‐3p remarkably increased the number of yellow puncta, with only a slight influence on RFP‐only puncta, suggesting that miR‐143‐3p inhibited the lysosomal processing of autophagosomes. In conclusion, miR‐143‐3p dampened lysosomal clearance and reduced autophagic degradation of CAMs in BMECs.

### 2.5 miR‐143‐3p Induced the Elevation of CAMs Through Targeting ATP6V1A

To elucidate the molecular mechanism underlying the dampening effect of miR‐143‐3p on lysosomal clearance, the target genes of miR‐143‐3p were predicted using various databases, including TargetScan, miRanda, miRDB, and miRWalk. Only candidate genes that appeared in all databases were considered (Figure 3F), and subsequently, an overlap analysis was conducted with genes associated with lysosomes in the Genecard Database (Figure 3G). From these analyses, two predicted targets, namely ATP6V1A and RNF152, were identified. Luciferase reporter assays revealed a significant reduction in ATP6V1A‐3′UTR‐driven luciferase activity upon transfection with miR‐143‐3p minics. In contrast, no notable change was observed when the mutated ATP6V1A 3′UTR was transfected with miR‐143‐3p minics (Figure S3A, Supporting Information). Moreover, WB analysis demonstrated that miR‐143‐3p minics significantly decreased the protein level of ATP6V1A (Figure S3C,D, Supporting Information). These findings strongly suggest that ATP6V1A is a direct and functional target of miR‐143‐3p. The target sites in the 3′UTR of ATP6V1A by miR‐143‐3p are highly conserved across different mammals (Figure 3H). However, no positive regulatory relationship was observed between miR‐143‐3p and RNF152 (Figure S3B, Supporting Information).

ATP6V1A, an ATPase H+ transporting V1 lysosomal subunit A, is a membrane protein that utilizes ATPase to acidify the lysosome. This acidification process is crucial for lysosomal hydrolytic activity and autophagy.^[^ ^40^, ^44^, ^45^ ^]^ Consistent with the downregulation of ATP6V1A, overexpression of miR‐143‐3p resulted in lysosomal de‐acidification (Figure 3I). Moreover, this de‐acidification was accompanied by decreased enzymatic activities of CTSB and CTSD, which are two major lysosomal proteases (Figure 3J). Importantly, treatment with EN6, an ATP6V1A agonist, promoted lysosomal acidification, restored lysosomal enzyme activities, and mitigated the impaired autophagic flux induced by miR‐143‐3p (Figure 3K–M). Meanwhile, EN6 treatment significantly reversed the elevation of CAMs protein expression induced by miR‐143‐3p both in hCMEC/D3 (Figure 3N) and bEnd.3 cells (Figure S4A,B; Supporting Information). Altogether, these results suggested that miR‐143‐3p impairs the autophagic degradation of CAMs through targeting ATP6V1A.

### 2.6 Endothelial‐Specific ATP6V1A Knockout Increased CAMs Expression and Neutrophils Infiltration after ICH in Mice

**Figure 4:**
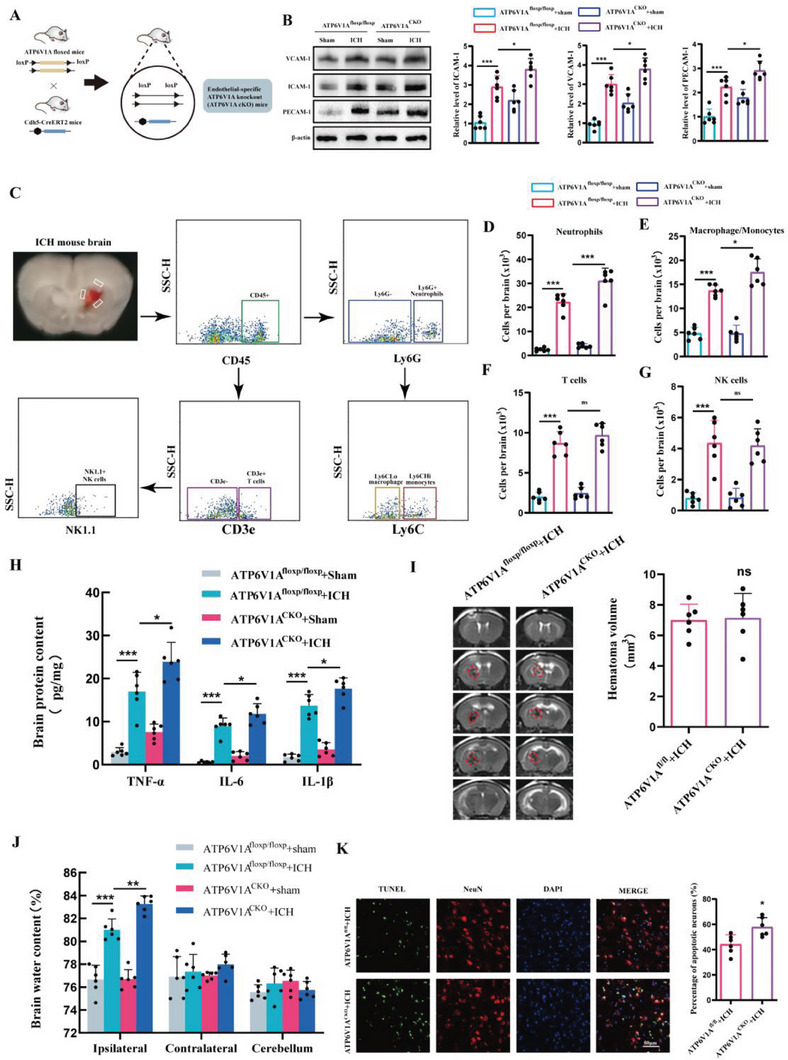
Endothelial‐specific ATP6V1A knockout increased CAMs expression and neutrophil infiltration after ICH in mice. A) Establishment of endothelial‐specific ATP6V1A knockout mice. B) Western blot assay of the expression of CAMs including VCAM‐1, PECAM‐1, and ICAM‐1 at 24 h post‐ICH in ATP6V1A ^fl/fl^ and ATP6V1A ^CKO^ mice (*n =* 6 per group, one‐way ANOVA). C) Representative gating strategy for flow cytometric analysis of immune cells in the brain at 24 h post‐ICH in ATP6V1A ^fl/fl^ and ATP6V1A ^CKO^ mice. D–G) Cell surface antibodies were used to identify different cell types including D) Ly6G^+^ neutrophils, E) Ly6G^−^ Ly6C macrophage/monocytes, F) CD3e+ T cells, G) NK1.1+ NK cells (*n =* 6 per group, one‐way ANOVA). H) The expression of IL‐1β, IL‐6, and TNF‐ɑ in the brain at 24 h after ICH in ATP6V1A ^fl/fl^ and ATP6V1A ^CKO^ mice was analyzed by ELISA (*n =* 6 mice per group, one‐way ANOVA). I) MRI images and quantification of hematoma at 24 h after ICH in ATP6V1A ^fl/fl^ and ATP6V1A ^CKO^ mice. Red lines delineate the lesion area. (*n =* 6 per group, one‐way ANOVA). J) Brain water content at 24 h after ICH in ATP6V1A ^fl/fl^ and ATP6V1A ^CKO^ mice (*n =* 6 per group, one‐way ANOVA). K) Representative images and statistical analysis of TUNEL‐positive cells in mice brain sections. Data are represented as the percentage of TUNEL‐positive cells (*n =* 6 per group, Student's t‐test). Scale bar: 50 µm. Data are presented as means ± SD. *p < 0.05, **p < 0.01, ***p < 0.001.

Until now, there have been no reports on the role of ATP6V1A in post‐ICH conditions. Therefore, we conducted several in vivo experiments to identify the changes in endothelial ATP6V1A after ICH and its role in the progression of secondary brain injury. WB results showed a significant decrease in the expression of ATP6V1A in isolated BMECs after ICH (Figure S5A, B, Supporting Information). We then successfully generated endothelial‐specific ATP6V1A knockout mice (ATP6V1A^CKO^) by crossing ATP6V1A‐floxed mice with Cdh5 creERT2 mice, followed by tamoxifen administration (**Figure** **4**A; Figure S5C,D, Supporting Information). On day 1 after ICH, the expression of VCAM‐1, ICAM‐1, and PECAM‐1 were significantly up regulated in the peri‐hematoma region when compared to the sham group. Furthermore, the expression levels of these molecules were even higher in the ATP6V1A^CKO^ ICH group compared to the ATP6V1A^fl/fl^ ICH group (Figure 4B).

Given the critical role of CAMs in TEM of peripheral immune cells and neuroinflammation, we then investigate whether endothelial‐specific ATP6V1A knockout affects brain infiltration of peripheral immune cells. Flow cytometry analysis was performed to quantify immune cell populations in the brain 1 day post‐ICH (Figure 4C). Accompanied by the higher expression of CAMs, the ATP6V1A ^CKO^ ICH mice showed a significant increase in neutrophils in the brain compared to the ATP6V1A ^fl/fl^ ICH mice (Figure 4D). Additionally, there was a slight increase in macrophage/monocyte cell numbers in the ATP6V1A ^CKO^ ICH mice, but no significant differences were observed in T and NK cell subsets when compared to the ATP6V1A ^fl/fl^ ICH mice (Figure 4E–G). This could be due to the fact that neutrophils are the first cells to respond and migrate from the peripheral blood to the brain after a stroke in the acute period.^[^ ^46^, ^47^ ^]^ To assess whether the significant differences in neutrophils were due to higher levels of circulating neutrophils in ATP6V1A ^CKO^ mice, blood samples were collected and circulating neutrophils were compared (Figure S6A, Supporting Information). The neutrophil counts in the peripheral blood were comparable between ATP6V1A ^fl/fl^ ICH and ATP6V1A ^CKO^ ICH mice (Figure S6B, Supporting Information). These results indicate that the higher infiltration of neutrophils in brain tissue of ATP6V1A ^CKO^ ICH mice than ATP6V1A ^fl/fl^ ICH mice is not due to higher levels of circulating neutrophils, but rather enhanced efficiency of neutrophil migration into the brain. Furthermore, quantification of pro‐inflammatory cytokines IL‐1β, TNF‐α, and IL‐6 revealed a more pronounced neuroinflammation in ATP6V1A ^CKO^ ICH mice compared to ATP6V1A ^fl/fl^ ICH mice (Figure 4H). Consistent with these findings, although MRI results indicated that endothelial cell‐specific ATP6V1A deletion didn't affect the volume of hematoma at 24 h post‐ICH (Figure 4I), it indeed resulted in a more severe extent of brain edema (Figure 4J) and neuronal apoptosis (Figure 4K). In summary, our study demonstrates that endothelial‐specific ATP6V1A knockout contributes to enhanced expression of CAMs, increased neutrophil infiltration, and exacerbation of secondary brain injury after ICH.

### 2.7 VCAM‐1 targeting EVs Effectively Deliver miR‐143‐3p Inhibitor to Pathological BMECs

**Figure 5:**
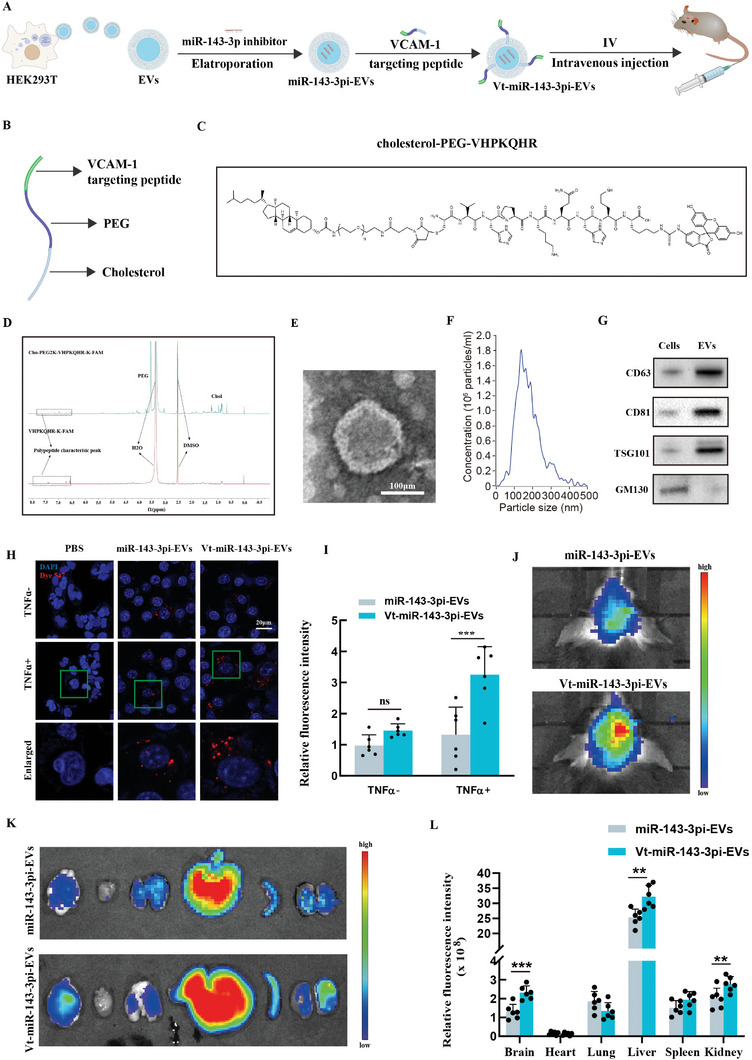
VCAM‐1‐targeting EVs effectively deliver miRNA‐143‐3p inhibitor into pathological BMECs. A) Schematic diagram showing the production of VCAM‐1‐targeting, miR‐143‐3p inhibitor‐encapsulated EVs (Vt‐miR‐143‐3pi‐EVs). B) The illustration of cholesterol‐modified VCAM‐1‐targeting peptide. C) Chemical structure of cholesterol‐modified VCAM‐1‐targeting peptide. D) ^1^H NMR spectrum of VCAM‐1‐targeting peptide and cholesterol‐modified VCAM‐1‐targeting peptide. E) Transmission electron microscopy analysis and F) nanoparticle tracking analysis of Vt‐miR‐143‐3pi‐EVs. G) Western blot analysis of several EVs marker proteins in Vt‐miR‐143‐3pi‐EVs. (H‐I) Representative confocal images and quantitative analyses of the cellular uptake of Dye 547‐ labeled miRNA inhibitor delivered by nontargeting EVs or VCAM1‐targeting EVs in vehicle or TNFɑ‐treated hCMEC/D3 cells (*n =* 6 per group, One‐way ANOVA). Scale bar: 20 µm. J–L) Live fluorescence imaging and analysis of the distribution of DiR‐labelled nontargeting miR‐143‐3pi‐EVs or Vt‐miR‐143‐3pi‐EVs in ICH mice 24 h after intravenous injection. (*n =* 6 per group, Student's t‐test). Data are presented as means ± SD. *p < 0.05, **p < 0.01, ***p < 0.001.

The aforementioned research results suggest a causal relationship between EVs‐miR‐143‐3p and brain endothelial dysfunction following ICH. Subsequently, we explored nucleotide‐based therapies targeting miR‐143‐3p for ICH treatment. Due to the susceptibility of miRNA inhibitors to degradation, we overcame this challenge by encapsulating the miR‐143‐3p inhibitor into EVs, which are natural vesicles that prevent the contents from degradation and exhibit better biocompatibility compared with artificial materials (**Figure** **5**A). Simultaneously, we explored methods to enhance the targeting ability of EVs toward the pathological BMECs. Notably, CAMs are highly expressed on injured BMECs after ICH, particularly VCAM‐1, which is considered a specific surface marker of pathological endothelial cells^[^ ^.48^ ^]^ Hence, we modified the surface of EVs by attaching a targeting peptide (VHPKQHR) that was reported to recognize VCAM‐1^[^ ^49^, ^50^ ^]^ (Figure 5A). The first step involved linking VCAM‐1‐targeting peptide and cholesterol‐PEG‐maleimide via thiol‐maleimide cross‐linking to synthesize the cholesterol‐modified VCAM‐1‐targeting peptide (Figure 5B,C). The successful conjugation of VCAM‐1‐targeting peptide to cholesterol‐PEG‐maleimide was confirmed by the chemical structure and 1H NMR spectrum (Figure 5D). Given that cholesterol exhibits automatic insertion into the cell membrane, EVs functionalization was achieved via co‐incubation with cholesterol‐modified VCAM‐1‐targeting peptide. TEM and NTA analysis unveiled that VCAM‐1‐targeting, miR‐143‐3p inhibitor‐loaded EVs (Vt‐miR‐143‐3pi‐EVs) exhibited a 100–200 nm double‐layered vesicular structure (Figure 5E,F). WB analysis confirmed that Vt‐miR‐143‐3pi‐EVs were positive for EV marker proteins including CD63, CD81, and TSG101, but negative for the cis‐Golgi marker protein, GM130 (Figure 5G).

We then carried out in vitro studies to evaluate the effectiveness of Vt‐miR‐143‐3pi‐EVs in delivering miR‐143‐3p inhibitors to pathological BMECs. For visualization of cellular uptake, the miR‐143‐3p inhibitor was labeled with Dye 547. TNFα treatment was employed to induce the activation of hCMEC/D3 cells and expression of VCAM‐1. Subsequently, vehicle or TNFɑ‐treated hCMEC/D3 cells were incubated with Dye 547‐labeled miR‐143‐3p inhibitor, delivered either by EVs or Vt‐EVs. Confocal microscopy analysis revealed limited uptake of the Dye 547‐labeled miR‐143‐3p inhibitor when delivered by non‐targeting EVs in both control and TNFɑ‐stimulated hCMEC/D3 cells. In contrast, a substantial uptake of the Dye 547‐labeled miR‐143‐3p inhibitor was observed when delivered by Vt‐EVs in TNFɑ‐treated hCMEC/D3 cells, while quiescent cells showed no significant uptake (Figure 5H,I).

Then, the biodistribution of Dir‐labeled miR‐143‐3pi‐EVs or Vt‐miR‐143‐3pi‐EVs in ICH mice was tracked in vivo after administering at a dosage of 200 µg via intravenous injection. An in vivo imaging system (IVIS) was employed for this purpose. Figure 5J illustrates the subsequent fluorescence intensity in the ICH mouse brain observed 24 h post‐injection of miR‐143‐3pi‐EVs or Vt‐miR‐143‐3pi‐EVs. And quantification of fluorescence in various organs (brain, heart, liver, spleen, lung, and kidney) showed the liver exhibiting the most substantial accumulation. Notably, the fluorescence intensity in brain regions was considerably higher for Vt‐miR‐143‐3pi‐EVs treatment compared to miR‐143‐3pi‐EVs treatment (Figure 5K,L). This finding further emphasizes the functional impact of the VCAM‐1‐targeting peptide utilized to modify the EVs. To further validate the biological function of Vt‐EVs loaded with miR‐143‐3p inhibitor, we measured the protein expression level of ATP6V1A in isolated BMECs and observed a significant increase of ATP6V1A protein expression in ICH following Vt‐miR‐143‐3pi‐EVs administration (Figure S5F,G, Supporting Information). In summary, VCAM‐1‐targeting peptide‐engineered EVs effectively deliver miRNA‐143‐3p inhibitor to pathological BMECs and prevent the decrease of ATP6V1A expression in ICH.

### 2.8 Therapeutic Effects of VCAM‐1–Targeting, miR‐143‐3p Inhibitor‐Encapsulated EVs on ICH

**Figure 6:**
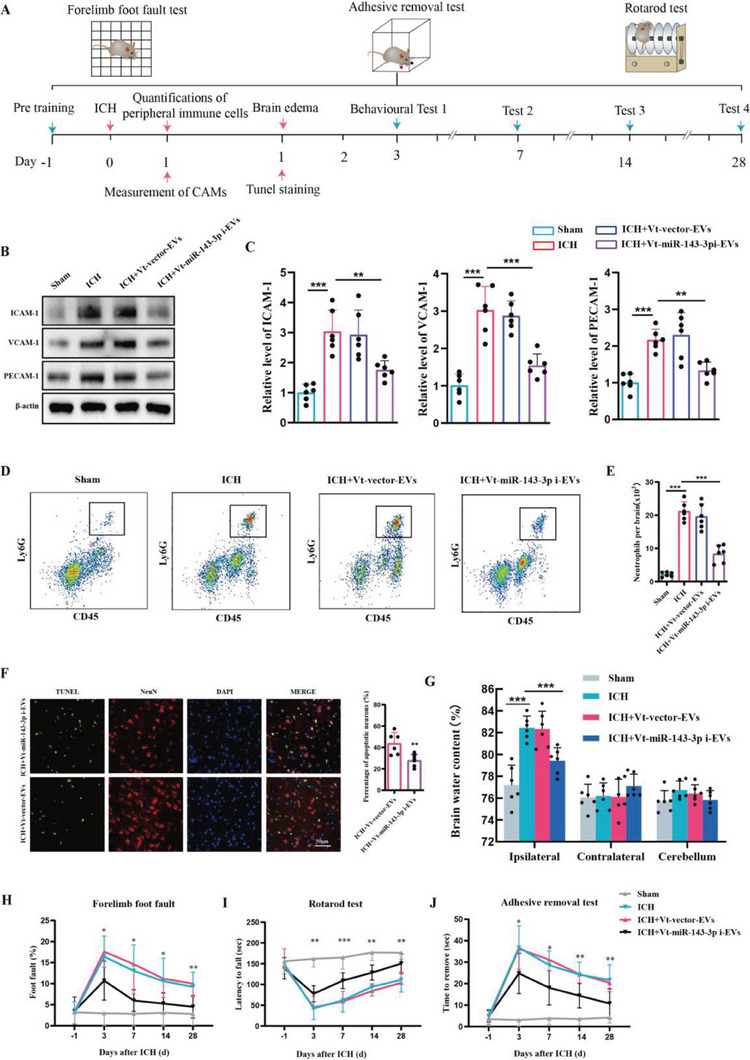
Therapeutic effects of VCAM‐1‐targeting, miR‐143‐3p inhibitor‐encapsulated EVs on ICH in mice. A) Experimental schematic. B,C) Western blot assay of the effects of Vt‐miR‐143‐3pi‐Evs on the expression of CAMs including ICAM‐1, VCAM‐1, and PECAM‐1 in isolated BMECs at 24 h post‐ICH in mice (*n =* 6 per group, one‐way ANOVA). D,E) Flow cytometric analysis of the effects of Vt‐miR‐143‐3pi‐Evs on the infiltration of Ly6G+ neutrophils in the brain at day 1 post‐ICH in mice (*n =* 6 per group, one‐way ANOVA). F) Representative images and statistical analysis of TUNEL‐positive cells in mice brain sections at 24 h post‐ICH in each group mice (*n =* 6 per group, Student's t‐test). Data are represented as the percentage of TUNEL‐positive cells. Scale bar: 50 µm. G) Measurement of brain water content at 24 h after ICH in each group mice (*n =* 6 per group, one‐way ANOVA). H–J) Several behavioral tests including a foot fault test, rotarod test, and adhesive test were conducted to determine the neurological function of mice (*n =* 12 per group, two‐way ANOVA). Data are presented as means ± SD. *p < 0.05, **p < 0.01, ***p < 0.001.

Subsequently, we examined the potential benefits of Vt‐miR‐143‐3pi‐EVs on brain recovery upon ICH. WB analysis revealed that the elevated expression of CAMs induced by ICH was significantly down‐regulated following Vt‐miR‐143‐3pi‐EVs treatment (**Figure** **6**B,C). Additionally, flow cytometry analysis demonstrated that the delivery of miR‐143‐3p inhibitor via Vt‐EVs significantly alleviated neutrophil infiltration in brain (Figure 6D,E), which was accompanied by a reduction in neuronal apoptosis (Figure 6F) and brain edema (Figure 6G). Then, several behavioral tests were conducted to investigate whether neurological deficits in ICH could be alleviated following Vt‐miR‐143‐3pi‐EVs treatment. As illustrated in Figure 6H, animals administered Vt‐miR‐143‐3pi‐EVs exhibited improved performance with reduced foot faults at day 3, 7, 14, and 28 after ICH surgery, as compared to the nontargeting EVs treated group in a foot fault test. The rotarod test and adhesive removal test yielded comparable outcomes (Figure 6I,J), thus supporting the therapeutic effect of the aforementioned treatment.

### 2.9 The Detrimental Role of EVs‐miR‐143‐3p in TBI and the Therapeutic Potential of Vt‐miR‐143‐3pi‐EVs Treatment

**Figure 7:**
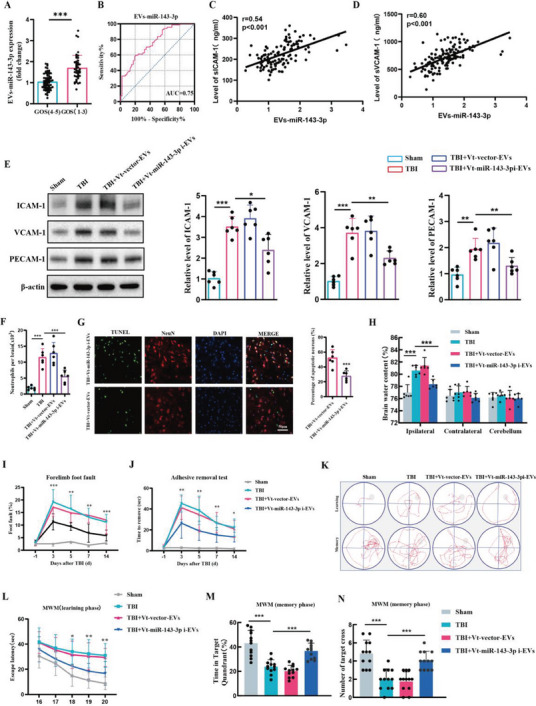
The detrimental role of EVs‐miR‐143‐3p in TBI and the therapeutic effects of Vt‐miR‐143‐3pi‐EVs treatment. A) Relative plasma EVs‐miR‐143‐3p level on the 1st day in TBI patients with poor (GOS 1–3) outcomes versus good (GOS 4–5) outcomes 6 months later. *n =* 67 in good outcome group and *n =* 46 in poor outcome group. GOS:Glasgow Outcome Scale. B) ROC curve for individual serum EVs‐miR‐143‐3p level on day 1 to separate poor (GOS 1–3) outcomes versus good (GOS 4–5) outcomes 6 months later. C,D) Pearson analyses of the correlations of EVs‐miR‐143‐3p with the serum levels of s‐VCAM‐1, and s‐ICAM‐1 within 24 h after TBI onset in patients (*n =* 113). E) Western blot assay of the effects of Vt‐miR‐143‐3pi‐EVs on the expression of CAMs including ICAM‐1, VCAM‐1, and PECAM‐1 in BMECs at 24 h post‐TBI in each group mice (*n =* 6 per group, one‐way ANOVA). F) Flow cytometric analysis of the effects of Vt‐miR‐143‐3pi‐EVs on the infiltration of Ly6G+ neutrophils in the brain at day 1 post‐TBI in mice (*n =* 6 per group, one‐way ANOVA). G) Representative images and statistical analysis of TUNEL‐positive cells in mice brain sections at 24 h post‐TBI in each group mice (*n =* 6 per group, Student's t‐test). Data are represented as the percentage of TUNEL‐positive cells. Scale bar: 50 µm. H) Measurement of brain water content at 24 h after TBI in each group mice (*n =* 6 per group, one‐way ANOVA). I–N) Several behavioral tests including foot fault test, adhesive test, and Morris water maze (MWM) test were conducted to determine the neurological function of mice (*n =* 12 per group, I, J, and L for two‐way ANOVA, M for one‐way ANOVA). Data are presented as means ± SD. *p < 0.05, **p < 0.01, ***p < 0.001.

After the onset of TBI, another prevalent form of acute brain injury, a similar pathological process of secondary brain damage accompanies it, including the expression of inflammatory CAMs and the infiltration of neutrophils. Hence, we further validate our aforementioned findings in ICH through the utilization of TBI patients and a TBI murine model. First, 113 patients with TBI were included to evaluate whether the level of EV‐miR‐143‐3p was associated with neurological outcomes. We found patients with poor outcomes (6 months GOS score was 1–3) had significantly higher plasma EVs‐miR‐143‐3p levels than those with favorable outcomes (6 months GOS score was 4–5) (**Figure** **7**A). Multivariate logistic regression analysis confirmed plasma EVs‐miR‐143‐3p level as an independent risk factor for TBI patients’ neurological outcomes (Table S1, Supporting Information). ROC curve analysis indicated that EVs‐miR‐143‐3p level possessed a great ability to predict TBI neurological outcomes (Figure 7B). Meanwhile, the level of EVs‐miR‐143‐3p was found to be positively correlated with the plasma levels of sICAM‐1 and sVCAM‐1 in TBI patients (Figure 7C,D).

Subsequently, the TBI murine model was also employed. It was discovered that compared with sham group mice, the expressions of CAMs were remarkably up‐regulated in TBI group mice, which were substantially reduced following Vt‐miR‐143‐3pi‐EVs treatment (Figure 7E). It also showed that Vt‐miR‐143‐3pi‐EVs treatment significantly inhibited neutrophil infiltration (Figure 7F), reduced neural apoptosis (Figure 7G), and attenuated brain edema (Figure 7H) in TBI. Foot fault test and adhesive test indicated that Vt‐miR‐143‐3pi‐EVs treatment resulted in improved sensorimotor functionality following TBI (Figure 7I,J). Meanwhile, previous studies indicated that learning and memory deficits are also prominent after TBI.^[^ ^51^, ^52^ ^]^ We then performed MWM experiments to test if TBI‐induced cognitive function impairments can be attenuated through Vt‐miR‐143‐3pi‐EVs treatment. In the learning phase, compared to sham group mice, the mean escape latency was longer in TBI group mice. In the spatial probe trial, the TBI group mice spent less time in the target quadrant and had fewer platform crossings than those from the sham group. Importantly, the above parameters were improved after Vt‐miR‐143‐3pi‐EVs treatment (Figures 7K–N). Hence, the Morris water maze test indicated a significant improvement in spatial learning and memory performance after Vt‐miR‐143‐3pi‐EVs treatment in TBI mice. In conclusion, we have ascertained that VCAM‐1‐targeting, miR‐143‐3p inhibitor‐encapsulated EVs hold immense promise as a therapy for acute brain injury.

## 3 Discussion

The significant involvement of EVs in the progression of various human diseases, including central nervous system (CNS) disorders, has been increasingly elucidated in recent years. By screening clinical blood samples from ICH patients, we identified that the level of EVs‐miR‐143‐3p is closely correlated with the progression of perihematomal edema and neurological outcomes. Further research revealed that the increased EVs‐miR‐143‐3p after ICH is mainly secreted by activated astrocytes and can be absorbed by BMECs, where it blocks the autophagic degradation of several key CAMs by targeting ATP6V1A. The increased cell surface expression of CAMs promotes excessive neutrophil TEM, leading to the worsening of brain injury. Hence, we engineered EVs that display VCAM‐1‐targeting peptide on the surface to selectively deliver miR‐143‐3p inhibitor to pathological BMECs, which effectively restores the barrier function of BMECs, blocks the neutrophil mitigation, and prevents secondary brain injury in both ICH and TBI mouse models. In summary, we have revealed a novel role of EV‐miR‐143‐3p in regulating endothelial barrier properties and presented a potentially widely applicable nucleotide drug delivery technology targeting pathological BMECs after acute brain injury.

EVs are diminutive membranous entities secreted by cells, encapsulating a diverse range of biomolecules including proteins, lipids, and nucleic acids. One of the most auspicious applications of EVs is their potential to serve as biomarkers for various diseases. Furthermore, EVs have been found to contribute to the development and progression of numerous ailments, rendering them an attractive target for therapeutic intervention. In this study, we employed an unbiased approach to identify elevated levels of EVs‐miR‐143‐3p in patients with ICH, displaying a strong negative correlation with functional recovery. This indicated that the level of EVs‐miR‐143‐3p may be a promising indicator of outcomes in patients with ICH. Meanwhile, it is well‐established that miR‐143‐3p is a brain‐specific miRNA. The heightened concentration of EVs‐miR‐143‐3p in the plasma of ICH patients may indicate a significant alteration in the levels of EVs‐miR‐143‐3p in the brain, which has been substantiated in the ICH mouse model. Furthermore, a sequence of in vitro and in vivo experiments has revealed that the increased levels of EVs‐miR‐143‐3p post‐ICH are primarily secreted by activated astrocytes and can be transferred to BMECs. Although the up‐regulated level of miR‐143‐3p in BMECs does not affect their viability, they do cause endothelial barrier dysfunction, particularly with regard to the increased expression levels of a series of CAMs, including VCAM‐1, ICAM‐1, and PECAM‐1. Experimental and clinical evidence unequivocally reveal a substantial upsurge in CAMs after the occurrence of stroke. CAMs are indispensable for the entrance of peripheral immune cells into cerebral tissue. Furthermore, meticulous clinical investigations have consistently indicated that heightened CAM levels in both cerebrospinal fluid (CSF) and serum of individuals afflicted with acute stroke are incontrovertibly linked to an unfavorable prognosis.^[^ ^53^, ^54^ ^]^

In the context of elucidating the mechanistic pathway through which miR‐143‐3p induced the upregulation of adhesion molecules, we initially explored the possibility of direct miRNA‐mRNA interactions between miR‐143‐3p and the transcripts encoding VCAM‐1, ICAM‐1, and PECAM‐1. However, in silico analysis revealed no conclusive evidence supporting such interactions. Furthermore, we observed no discernible disparity in mRNA expression levels of these molecules, irrespective of miR‐143‐3p overexpression. Consequently, we postulate that the elevated levels of adhesion molecules may be attributed to aberrant protein degradation and turnover. It is noteworthy that previous investigations have highlighted the pivotal role played by the autophagy‐lysosome degradation pathway in maintaining CAM expression levels. Nourshargh et al. demonstrated that non‐canonical autophagy modulates intracellular trafficking and degradation of PECAM‐1 in endothelial cells^[^ ^.55^ ^]^ Similarly, Piette et al revealed that the decreased expressions of ICAM‐1 and VCAM‐1 in endothelial cells, subsequent to photodynamic therapy, can be attributed to the degradation of these molecules by lysosomal proteases^[^ ^.56^ ^]^ Herein, we found that the transfer of miR‐143‐3p into BMECs can lead to a decrease in ATP6V1A levels, which causes lysosomal de‐acidification and a reduction in lysosomal hydrolytic capacity. This impaired lysosomal function hinders the autophagic degradation of these CAMs. However, treatment with an ATP6V1A activator can reverse the effects of miR‐143‐3p overexpression on BMECs lysosomal functions and protein levels of CAMs.

It is widely recognized that enhanced CAMs promote leukocyte TEM, which is a critical step in leukocyte infiltration of tissues. Indeed, by using EC ATP6V1A^CKO^ mice, we observed that accompanied by the increase in CAMs after ICH, the degree of neutrophil brain infiltration is also more pronounced. It is well‐known that neutrophils can exacerbate brain injury by releasing pro‐inflammatory cytokines, reactive oxygen species, and proteases, damaging neurons and the blood‐brain barrier. Neutrophils can also engage in interactions with other immune cells, including microglia and T cells, thereby amplifying the inflammatory response and promoting tissue damage. Consistent with increased neutrophil infiltration, we have observed a significant augmentation in oxidative stress‐induced damage, inflammatory response, and neuronal apoptosis within the brain. The mechanisms underlying neutrophil infiltration in acute brain injury are intricate and involve multiple signaling pathways, such as chemokines and adhesion molecules, as well as complement. Chemokines, such as CXCL1, CXCL2, and CCL2, can attract neutrophils to the injured brain tissue by binding to their receptors, such as CXCR2 and CCR2. Adhesion molecules, such as ICAM‐1, VCAM‐1, and E‐selectin, can facilitate the adhesion and transmigration of neutrophils across the blood‐brain barrier and into the brain parenchyma. Therefore, one of the central tenets of this study is that if we can elucidate the molecular mechanisms underlying the up‐regulation of adhesion molecules on endothelial cells and undertake targeted intervention, we can effectively alleviate the secondary brain injury mediated by neutrophil infiltration.

Given the association between abnormal EVs‐miRNA‐143‐3p level and endothelial barrier dysfunction, it may be a feasible therapeutic strategy to deliver therapeutic nucleotides, such as miRNA‐143‐3p inhibitor, to pathological endothelium. Compared to peripheral organs, targeted drug delivery to the central nervous system (CNS) is often more challenging due to the presence of the blood‐brain barrier^[^ ^.57^ ^]^ EVs possess exceptional capabilities as gene‐delivery vehicles owing to their ability to sequester charged nucleotides. This unique property shields nucleic acids from nonspecific interactions and enzymatic degradation, rendering EVs highly valuable for therapeutic applications. Moreover, their remarkable biocompatibility further enhances their potential as gene carriers. Exploiting the versatility of gene modification techniques, specific ligands can be expressed on the EV membrane surface, facilitating targeted delivery to desired cell types. A notable example of this approach is demonstrated by Li et al.^[^ ^29^ ^]^ who successfully modified EVs derived from HEK293T cells with acetylcholine receptor (AChR)‐specific rabies virus glycoprotein (RVG) peptides. This modification enabled effective drug delivery across the blood‐brain barrier (BBB) for the treatment of brain diseases. In line with this concept, our study aimed to target pathological BMECs. Specifically, we focused on VCAM‐1, a prominent endothelial adhesion molecule that is up‐regulated in pathological endothelial cells, while remaining low in healthy endothelial cells. To achieve targeted delivery, we engineered EVs to display a VCAM‐1‐targeting peptide known as VHPKQHR. Our experimental findings unequivocally demonstrate the efficacy of this VCAM‐1‐targeting strategy in actively delivering miRNA inhibitors to pathological BMECs, consequently restoring endothelial barrier function.

The heightened expression of inflammatory CAMs and the infiltration of neutrophils are the shared pathological mechanisms underlying acute brain injuries, including ICH, ischemic stroke, subarachnoid hemorrhage (SAH), and TBI5,8,58,59. Given our encouraging findings in ICH, we sought to determine whether targeting EVs‐miRNA‐143‐3p could also be applicable to treating other types of acute brain injuries. Significantly, we recruited 113 TBI patients and identified the plasma level of EVs‐miR‐143‐3p as an independent prognostic factor for neurological outcomes in TBI patients. Moreover, we observed a positive correlation between the level of EVs‐miR‐143‐3p and plasma levels of sICAM‐1 and sVCAM‐1 in TBI patients. Additionally, in a TBI mouse model, treatment with Vt‐miR‐143‐3pi‐EVs significantly suppressed the expression of CAMs, neutrophil infiltration, neural apoptosis, and neurological deficits. These results suggest that targeting EVs‐miR‐143‐3p holds great potential as a therapeutic approach for acute brain injuries, particularly ICH and TBI. Furthermore, in future studies, we plan to further explore and validate this approach in other types of acute brain injuries, such as ischemic stroke and SAH.

However, our investigation is not without limitations that warrant attention. Firstly, our analysis of EVs‐miR‐143‐3p level was limited to plasma samples collected solely within day 1 post‐ICH, and thus the accuracy of the reported association may be impacted by a lack of data over a prolonged follow‐up period. Secondly, we did not conduct a functional assay on miR‐125‐5p, as no significant discrepancies in extracellular vesicular miR‐125‐5p levels were observed in the plasma of ICH mice. Given that extracellular vesicular miR‐125‐5p levels in the plasma of patients were linked to patient outcomes, the role of EVs‐miR‐125‐5p in secondary injury following ICH necessitates exploration. Thirdly, recent research has highlighted sex differences in response to acute brain injury, including differences in inflammatory responses, neuronal damage, and functional recovery. For example, previous study has shown that female animals often exhibit a more mitigated neuroinflammation and better recovery following traumatic brain injury compared to males^[^ ^.60^ ^]^ These differences may be attributed to hormonal, genetic, and epigenetic factors. Understanding potential sexual effects could inform personalized treatment strategies for brain injury. Future studies should consider including both male and female animals to investigate any potential sex differences in the therapeutic effects.

To summarize, we have explicated the causal role of elevated EVs‐miR‐143‐3p in the dysfunction of endothelial cell barriers, as well as the secondary brain injuries mediated by immune cell infiltration. Furthermore, we have established the therapeutic efficacy of VCAM‐1‐targeting, miR‐143‐3p inhibitor‐encapsulated EVs in promoting functional recovery following acute brain injuries.

## 4 Experimental Section

### Human Blood Samples

Human research in this study was approved by the Ethics Committee of Tangdu Hospital, the Air Force Military Medical University (TD‐202105‐13). Informed consent was signed by participants or their legally authorized representatives. In the screening phase, 20 healthy controls and 25 patients with ICH were included to determine the differentially expressed EVs‐miRNAs induced by ICH insult. In the validation cohort, 45 healthy controls and 76 patients with ICH were included for further analysis. Participants were aged 18–65 years older. Patients diagnosed with active malignancy, acute infections, systemic inflammatory diseases, diabetes, or psychiatric diseases were excluded. Neurological outcomes were assessed at 6 months after ICH using the modified Rankin scale (mRS) score, categorized as either good (mRS = 0‐2) or poor (mRS = 3‐6) outcomes. Within 24 h of initial symptoms, blood samples were collected from patients from the cubital vein and were buffered with ethylenediaminetetraacetic acid (EDTA). Blood samples were first centrifuged at 2000*×g* for 10 min, followed by centrifugation at 10 000*×g* for 2 min to remove platelets. Plasma samples were then stored at −80 °C for further analysis. Meanwhile, this study enrolled 113 TBI patients who were 18 – 65 years old within 24 h of onset of symptoms. Exclusion criteria and blood sample preparation were conducted as described above. Neurological outcomes were assessed at 6 months after TBI using the Glasgow Outcome Scale (GOS) score, categorized as either poor (GOS = 1–3) or good (GOS = 4–5) outcomes^[^ ^.61^ ^]^

### Animals

Animal experiments in this study were approved by the Animal Experiment Center of Air Force Military Medical University (IACUC‐20210555) and performed in accordance with the ARRIVE guidelines hosted on the NC3Rs website. C57BL/6J male mice (8–12 weeks) were obtained from the Animal Experiment Center of Air Force Military Medical University. Endothelial‐specific ATP6V1A knockout mice were constructed by cross‐breeding ATP6V1A floxed mice with Cdh5‐CreERT2 mice (both from Shanghai Model Organisms Center), followed by tamoxifen (75 mg k^−1^g, #S1238, Selleckchem, Houston, TX, USA) administration for five consecutive days. Mice are all housed in a temperature‐controlled animal facility with free access to food and water.

### ICH Mouse Model

Briefly, mice were fixed in a stereotactic apparatus under isoflurane anesthesia. Then, 0.05 U collagenase VII (#C0773, Sigma‐Aldrich, St. Louis, MO, USA) in 0.2 µL saline was prepared and injected stereotactically into the specified region (2.0 mm lateral and 0.4 mm forward to the bregma, 3.2 mm deep) for 5 min, followed by a waiting period of 5 min. Except for the collagenase injection, the sham group underwent the same procedure.

### TBI Mouse Model

TBI model mice were induced with a controlled cortical impact (CCI) device^[^ ^.62^ ^]^ Briefly, after isoflurane anesthesia, mice were fixed in a stereotaxic frame. In the area of 1.5 mm behind bregma and 1.5 mm lateral to the midline, a 3‐mm‐diameter spot was opened on the skull. An impactor piston tip of 2 mm was then installed perpendicular to the exposed cortex. The cortex was struck with a flat metal tip at a speed of 3 m ^−1^s, depth of 1.5 mm, and duration of 200 milliseconds. Sham mice underwent craniotomy without being injured.

### EVs Isolation

EVs from plasma or culture medium supernatant were isolated following previously described methods^[^ ^.21^ ^]^ The samples underwent centrifugation at 2000×g for 10 min and at 10 000×g for 30 min successively to remove cellular debris. The resulting supernatant was then subjected to a further centrifugation step at 100 000×g and 4 °C for 70 min to obtain the EVs. For EV purification, EVs were resuspended in sterile phosphate‐buffered saline (PBS) and underwent another round of centrifugation at 100 000×g and 4 °C for 70 min. Purified EVs were then resuspended in sterile PBS or RIPA buffer.

EVs from the mouse brain tissues were isolated, as described previously^[^ ^.63^ ^]^ Brain tissues were gently sliced and transferred to Hibernate‐E medium (Gibco, New York, USA) containing 75 U mL^−1^ collagenase type III (800 µL buffer was used per 100 mg brain tissue) for 20 min at 37 °C. Subsequently, the samples underwent sequential centrifugation at 300×g for 5 min, 2000×g for 10 min, and 10 000×g for 30 min at 4 °C. The resulting supernatant was filtered through a 0.22‐µm filter and layered on a triple sucrose cushion (0.6, 1.3, and 2.5 m). After centrifugation at 180 000×g and 4 °C for 3 h, fraction 2 was collected, diluted with PBS, and further centrifuged at 100 000*×g* under 4 °C for 70 min. Purified EVs were then resuspended in sterile PBS or RIPA buffer.

### Characterize of EVs

The concentration and size of EVs were determined with a PMX 110 ZetaView nanoparticle tracking analysis (NTA) system (Particle Metrix, Meerbusch, Germany). The 110‐nm polystyrene particles were used for calibration. Meanwhile, EVs were imaged with a transmission electron microscope (Japan Electron Optics Laboratory Company, Tokyo, Japan).

### Isolation of Brain Endothelial Cells

Brain endothelial cells were isolated following previously described methods^[^ ^.64^ ^]^ In brief, mice were euthanized and subjected to perfusion with PBS. Subsequently, their brains were extracted and dissociated utilizing the Neural Tissue Dissociation Kit (#130‐092‐628, Miltenyi Biotec, Germany) in accordance with the manufacturer's guidelines. Myelin Removal Beads (#130‐096‐733, Miltenyi Biotec, Germany) were then employed to eliminate myelin. endothelial cells were selectively enriched employing CD31b antibody‐coupled MicroBeads (#130‐097‐418, Miltenyi Biotec, Germany) following the manufacturer's instructions. The CD31b‐positive fraction was collected for further assessment.

### Quantitative Real‐Time PCR (qPCR)

First, total RNA was extracted using TRIzol reagent (Invitrogen, Carlsbad, CA, USA). RNA concentrations were confirmed using the NanoDrop Spectrophotometer (Thermo Scientific, USA). Then the reverse transcription of extracted RNAs were performed using The Mir‐XTM miRNA First‐Stand Synthesis Kit (Takara, Tokyo, Japan) following the manufacturer's instructions. A SYBR Premix Ex TaqTM II Kit (Takara, Tokyo, Japan) was then utilized for PCR amplification. Internal control for miRNA measurements was established using cel‐miR‐39. The 2−ΔΔCT method was employed for the relative quantification of miRNAs.

### Western Blotting

Total protein from brain tissues or cells was extracted and quantified. Proteins were separated using sodium dodecyl sulfate‐polyacrylamide gel electrophoresis (SDS‐PAGE), which were then transferred to polyvinylidene fluoride (PVDF) membranes (Millipore, Billerica, MA, USA). Subsequently, the membranes were blocked with 5% nonfat milk at room temperature for 2 h. To detect specific proteins, the membranes were incubated overnight at 4 °C with anti‐CD63 (25682‐1‐AP, Proteintech, Rosemont, Illinois, USA), anti‐GM130 (PA5‐95727, Invitrogen, Carlsbad, CA, USA), anti‐CD81 (27855‐1‐AP, Proteintech, Rosemont, Illinois, USA), anti‐TSG101 (14497‐1‐AP, Proteintech, Rosemont, Illinois, USA), anti‐ATP6V1A (ab199326, Abcam, Cambridge, UK), anti‐ICAM‐1 (16174‐1‐AP, Proteintech, Rosemont, Illinois, USA), anti‐PECAM‐1 (ab281583, Abcam, Cambridge, UK), anti‐VCAM‐1 (MA5‐31965, Invitrogen, Carlsbad, CA, USA), anti‐E‐selectin (20894‐1‐AP, Proteintech, Rosemont, Illinois, USA), anti‐β‐actin (20536‐1‐AP, Proteintech, Rosemont, Illinois, USA). Following three washes with TBST, the membranes were incubated with secondary antibodies for 2 h. After an additional three washes with TBST, the protein bands were detected and analyzed.

### MRI Analysis

Small‐animal 3T MRI scanner (Bruker MRI GmbH, Germany) was used to measure lesion volumes, as previously described^[^ ^.65^ ^]^ After anesthetized, mice were positioned on an MRI‐compatible holder. Cerebral structural data were acquired using a phased array mouse brain coil and radiofrequency surface. Three‐dimensional coronal and sagittal T2‐weighted (T2‐W) images were obtained for each mouse. Manual outlining of the lesions was performed, and the volumes (*V*) were calculated using the formula *V* = ∑ ((the long axis length in the lesion area in coronal monolayer perpendicular × width) × section thickness). Data were then analyzed by two independent investigators who were blinded to the groups.

### Neurobehavioral Tests

*Foot fault test*. Foot fault test was carried out to measure sensorimotor coordination during spontaneous locomotion. Mice were placed on a standard metal wire grid surface and recorded for 1 min. The number of foot faults and total steps were calculated. The results were presented as the percentage of foot faults over the total steps.

*Adhesive removal test*. The adhesive removal test was used to evaluate tactile responses and sensorimotor asymmetries. A 2 mm × 3 mm piece of adhesive tape was applied to the left paw (impaired side). The time required to remove the adhesive tape using the mouth was recorded. The observation period was limited to 120 s.

*Rotarod Test*. The rotarod test was employed to evaluate post‐ICH motor functions. Mice were placed on an apparatus consisting of a rotating rod with a gradual increase in speed from 4 to 40 rpm over a duration of 5 min. Each mouse underwent three trials on a given day, with a 5 min intertrial interval. The time taken by each mouse to fall off the rotating rod was meticulously recorded, and the mean values were calculated based on the three trials.

*Morris Water Maze (MWM)*. The MWM test was employed to evaluate the spatial learning and memory performance of the mice. Prior to any surgical intervention, the mice underwent a three‐day pre‐training phase to acclimatize to the testing environment and familiarize themselves with the location of the platform. Mice that failed to locate the platform on the final day of pre‐training were excluded from the subsequent formal testing procedures. During the learning phase, the mouse was gently placed into the pool and allowed to freely swim for a maximum of 1 min before finding the hidden platform. The time it took for the mouse to find the platform (escape latency) served as an indicator of spatial learning. Each trial concluded with the mouse either remaining on the platform or being manually placed on it if it failed to locate it within the designated time. Five consecutive days were dedicated to testing the mice. Following the learning phase, a memory phase was initiated by removing the platform. A single, 60 s probe trial was conducted to assess memory retention. During this trial, the percentage of time the mouse spent swimming in the goal quadrant and the number of target crosses were meticulously recorded and subsequently calculated.

### Cell Culture and Treatment

To culture primary astrocytes and microglia, neonatal C57BL/6 mice (P1) were sacrificed, and brain tissue was cut into pieces and trypsinized. Dissociated cells were resuspended in DMEM/F12 medium (Gibco, New York, USA) containing 10% fetal bovine serum (Gibco, New York, USA) and 1% penicillin/streptomycin (Sigma‐Aldrich, St. Louis, MO, USA), followed by incubation for 10 days. Microglia were isolated from mixed glial cells by shaking at 200 rpm for 3 h, resulting in purified astrocytes. To culture primary neurons, pregnant C57BL/6 mice were sacrificed on embryonic day 18. Embryos were collected, and the brain tissue was cut into pieces and trypsinized. Cells were placed in pre‐coated with poly‐l‐ornithine/laminin (Sigma‐Aldrich, St. Louis, MO, USA). Cells were then cultured in Neurobasal Medium (Gibco, New York, USA) containing L‐glutamine, B27 supplement, and penicillin/streptomycin for one week. NHA, HMC3, SY5Y, and bEnd.3 cell lines were cultured in DMEM (Gibco, New York, USA) containing 10% fetal bovine serum at 37 °C in an incubator at 95% humidity under 5% CO_2_. hCMEC/D3 cell line was cultured in ECM (Gibco, New York, USA) containing 10% fetal bovine serum at 37 °C in an incubator at 95% humidity under 5% CO_2_. Cells were exposed to 30 µM oxyhemoglobin (Sigma‐Aldrich, St. Louis, MO, USA) for 12 h to establish in vitro ICH model.^[^ ^66^, ^67^ ^]^

### EVs Labeling and Tracking

To evaluate EVs uptake, purified EVs were labeled with PKH26 (100 µM, Sigma‐Aldrich, St. Louis, MO, USA) following the manufacturer's instructions, and subsequently added to the culture medium of endothelial cells. After incubation for 12 h, cells were fixed and stained with DAPI for 10 min. After washing the excess dye using PBS, images were captured using a fluorescence microscope (A1 Si, Nikon, Tokyo, Japan).

### Luciferase Reporter Assays

Luciferase reporter assays were conducted in 96‐well plates to assess the effects of miR‐143‐3p minics or miR‐NC on the wild‐type or mutated ATP6V1A 3′‐UTR. HEK293T cells were co‐transfected with the respective constructs using Lipofectamine 2000 (Invitrogen, Carlsbad, CA, USA). After 24 h of transfection, the activities of Renilla luciferase and firefly luciferase were measured using a dual luciferase reporter assay kit (Promega, Madison, WI, USA).

### Lysosomal pH Measurement

Lysosomal pH was measured using a lysosomal pH detection kit (HR8268, Bio‐Rad Technologies, Hercules, CA, USA) according to the manufacturer's instructions. Cells were labeled with a P02 lysosomal pH fluorescence probe at 37°C for 5 min. The excess dye was washed using Hanks' Balanced Salt Solution. Fluorescence was detected using a microplate reader (excitation wavelength at 360–405 nm, emission wavelength at 525–550 nm). A calibration curve was generated for the fluorescence probe, and lysosomal pH was quantified.

### CTSD/CTSB Activity Assay

The CTSD/CTSB activity assay was performed as described previously^[^ ^.68^ ^]^ Cells were lysed using CTSD/CTSB cell lysis buffer (K143/K140, BioVision technologies, Milpitas, CA, USA) and centrifuged at 21 000×g for 5 min to remove cellular debris. The resulting supernatant was collected and added to a 96‐well plate, with the total volume adjusted to 50 µL using the CTSD/B cell lysis buffer. Subsequently, 50 µL of Master Assay Mix (K143/K140, BioVision technologies, Milpitas, CA, USA) was added to each well and incubated at 37 °C for 2 h. Fluorescence was then detected using a fluorometer equipped with a 328‐nm excitation/460‐nm emission filter for CTSD and a 400‐nm excitation/505‐nm emission filter for CTSB.

### Flow Cytometry

Flow cytometry analysis was conducted according to previously published methods^[^ ^.69^ ^]^ In brief, blood samples were collected and placed in ice‐cold PBS containing 5 mM EDTA. Ammonium chloride (ACK) solution was added to lyse the red blood cells. The samples were then washed three times with flow buffer, which consisted of PBS supplemented with 1% BSA. After that, the samples were incubated with antibodies on ice for 30 min, followed by three additional wash steps. Finally, the samples were suspended in 250 µL of flow buffer for FACS analysis. For brain sample preparation, mice were sacrificed and their brains were immediately collected in cold HBSS. The tissues were dissociated for an hour at 37 °C by digestion with Papain. The dispersed cells were then filtered through a 70 µm nylon mesh and collected by centrifugation. To further purify the cells, a 30% Percoll density gradient (17‐0891‐09, GE Healthcare, Milwaukee, WI, USA) was used. The cells were centrifuged at 900 g and 25 °C for 25 min, and the bottom fraction containing the isolated cells was collected. Subsequently, the cells were washed and resuspended in PBS containing 2% FBS. The cells were then stained with the following antibodies: APC‐eFluorTM780 anti‐mouse CD45 (47‐0451‐82, Thermo, Rockford, IL, USA), PE anti‐mouse Ly6G (16‐9668‐82, Thermo, Rockford, IL, USA), Pacific Blue anti‐mouse Ly6C (128064, Biolegend, San Diego, CA, USA), PE‐Cyanine7 anti‐mouse CD3e (25‐0031‐82, Thermo, Rockford, IL, USA), and APC anti‐mouse NK1.1 (17‐5941‐82, Thermo, Rockford, IL, USA). After incubation with the antibodies for 1 h, the cells were centrifuged and resuspended in PBS. All of the aforementioned data were acquired using a NovoCyte flow cytometer and analyzed using NovoExpress Software version.

### Construction of VCAM‐1–targeting, miR‐143‐3p Inhibitor Loaded EVs

The conjugation of VCAM‐1‐targeting peptide (VHPKQHRGC) to maleimide‐PEG‐cholesterol was performed through the thiol‐maleimide cross–linking reaction. Prior to conjugation, the C3‐thiol‐modified VCAM1‐targeting peptide were deprotected using 500 mM TCEP (pH 6.5). Subsequently, the deprotected peptide was coupled to maleimide‐PEG‐cholesterol at 4 °C for an overnight incubation, followed by dialysis to purify the peptide‐PEG‐cholesterol conjugate.

EVs were harvested from the HEK293T cells as mentioned above. To load miR‐143‐3p inhibitor into EVs, electroporation was performed based on a previous study^[^ ^,70^ ^]^ using 350 V/150 mF. The small EV/miRNA ratio was ≈400 µg of small EVs per 1 OD of miRNAs. Following electroporation, the unloaded miR‐143‐3p inhibitor attached to the EV surface was removed using RNase, followed by another round of EV isolation using the Exoquick kit (SBI System Biosciences, Palo Alto, CA, USA).

To conjugate 1.5E+11 particles (≈500 µg) of EVs with 100 µl of VCAM‐1‐targeting peptide‐PEG‐cholesterol conjugation, a shared incubation reaction was performed at 25 °C, 250 rpm for 3 h, followed by incubation at 4 °C for 24 h. The sample was transferred to a 100 kDa ultrafiltration tube (UFC8100, Millipore, Billerica, MA, USA) and the wash buffer was added to a final volume of 4 mL, resuspended, and centrifuged at 4000 g at room temperature for 20–30 min until the volume was ≈250 µL. This resuspension and centrifugation process was repeated twice, and the final sample volume was concentrated to ≈250 µL and transferred to an empty EP tube.

### Assessment of In Vitro Cellular Uptake and In Vivo Biodistribution Following EVs treatment

For cellular uptake assay, hCMEC/D3 cells were seeded onto a confocal dish. Subsequently, PKH26‐labeled EVs and VCAM‐1–targeting EVs (20 µg) were added to the medium and cultured for 24 h. The cells were fixed with 4% paraformaldehyde, and the nuclei were stained with DAPI (Beyotime, Shanghai, China). Images were captured using a fluorescence microscope (A1 Si, Nikon, Tokyo, Japan). To evaluate the biodistribution in vivo, 200 µg of Dir (Umibio, Shanghai, China) labeled EVs and VCAM‐1–targeting EVs were administered intravenously. After 24 h, the real‐time fluorescence intensity was analyzed using the IVIS imaging system (Xenogen, Alameda, CA, USA) with 620 nm excitation and 670 nm emission filters to detect the biodistribution.

### Statistical Analysis

Statistical analyses were performed using GraphPad Prism version 8.0 (GraphPad Software, Inc., La Jolla, CA, USA). A two‐tailed Student's t‐test was used to compare any two groups. For multi‐group comparisons, a one/two‐way analysis of variance (ANOVA) followed by a post hoc test was utilized. Correlations were calculated using Pearson's correlation coefficient test. All experimental subjects were randomly allocated to distinct experimental cohorts. All group allocation, data collection, and analysis were conducted in a blinded manner. The sample size for each experiment was indicated in the corresponding figure legend. No statistical methods were employed to predefine the sample sizes; however, the sample sizes were akin to those reported in previous scholarly works. Data were presented as mean ± standard deviation (SD) unless otherwise specified. Statistical significance was set at *p <* 0.05.

## Conflict of Interest

The authors declare no conflicts of interest.

## Author Contributions

X.W., H.L., Q.H., and J.W. contributed equally to this work. Y.Q. and S.G. designed the study. X.W., H.L., and Q.W. performed animal studies. Q.H., J.W., and T.W. performed the in vitro experiments. W.C., H.B., and L.L. were responsible for human clinical studies. J.L., P.Z., and L.H. helped to analyze miRNA‐seq data. Y.W. and C.G. helped to organize the figures. X.W., H.L., and Q.H. wrote the manuscript. Y.Q. and S.G. revised the manuscript.

## Supporting information

Supporting Information

## Data Availability

The data that support the findings of this study are available from the corresponding author upon reasonable request.
