# Supplementary material for: Astrocyte‐Derived Extracellular Vesicular miR‐143‐3p Dampens Autophagic Degradation of Endothelial Adhesion Molecules and Promotes Neutrophil Transendothelial Migration after Acute Brain Injury
Source: Adv Sci (Weinh). 2023 Dec 3;11(5):2305339. doi: 10.1002/advs.202305339 (PMC10837358; doi:10.1002/advs.202305339)
Supplement: Supplementary file 1 — Supporting Information [file ADVS-11-2305339-s001.pdf]

ADVANCED SCIENCE
Open Access

Supporting Information

for *Adv. Sci.*, DOI 10.1002/advs.202305339

Astrocyte-Derived Extracellular Vesicular miR-143-3p Dampens Autophagic Degradation of Endothelial Adhesion Molecules and Promotes Neutrophil Transendothelial Migration after Acute Brain Injury

Xun Wu, Haixiao Liu, Qing Hu, Jin Wang, Shenghao Zhang, Wenxing Cui, Yingwu Shi, Hao Bai, Jinpeng Zhou, Liying Han, Leiyang Li, Yang Wu, Jianing Luo, Tinghao Wang, Chengxuan Guo, Qiang Wang, Shunnan Ge and Yan Qu**

Supplementary Materials for

Astrocyte-Derived Extracellular Vesicular miR-143-3p Dampens Autophagic Degradation of Endothelial Adhesion Molecules and Promotes Neutrophil Transendothelial Migration after Acute Brain Injury

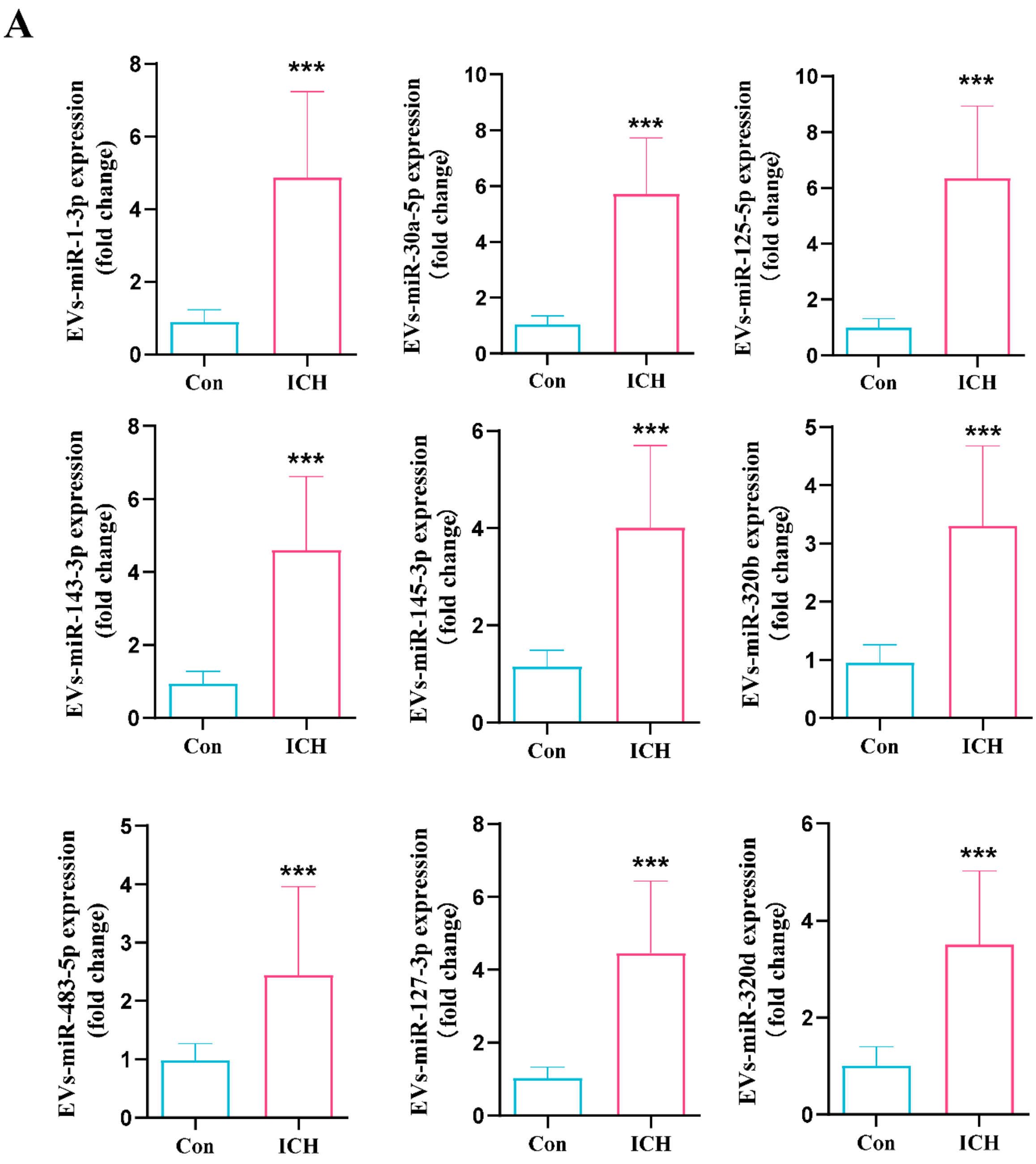

Figure S1. RT-qPCR analysis verify the differently expressed EVs-miRNAs between ICH patients and healthy controls (ICH = 76, control = 45; Student' s t-test). Data are expressed as mean ± SD. *P < 0.05; **P < 0.01; ***P < 0.001.

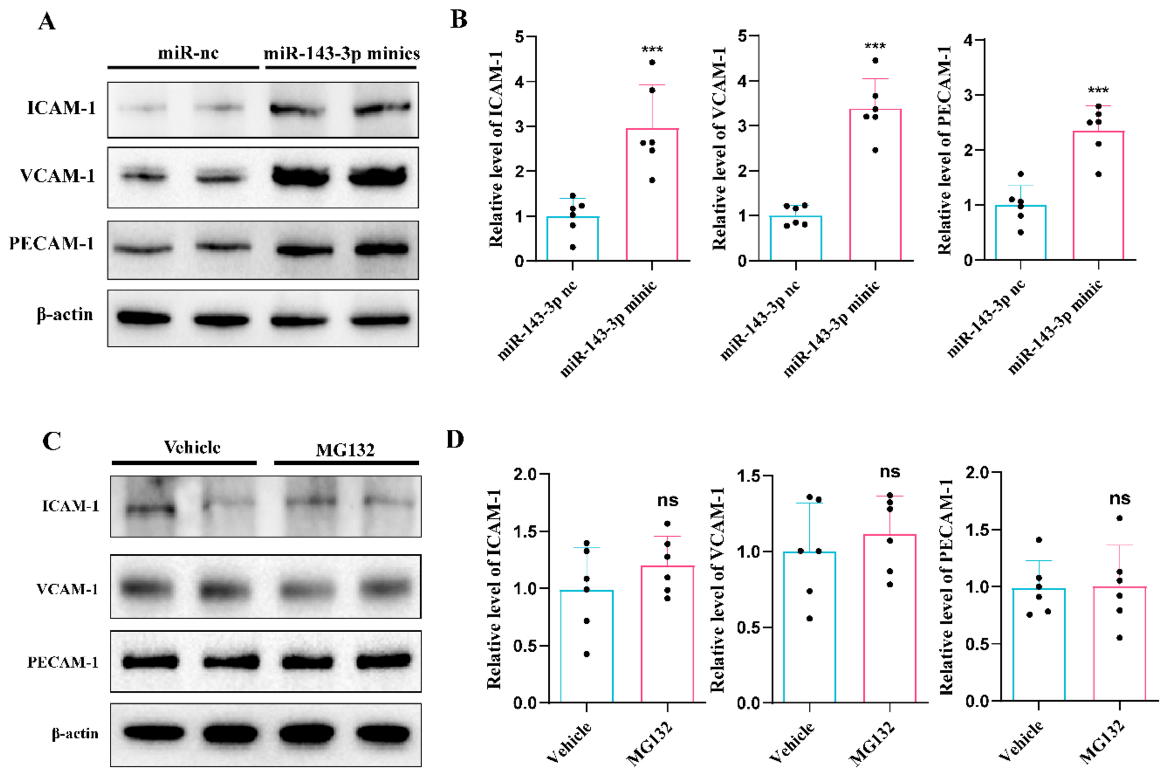

Figure S2. Detection of the levels of VCAM-1, ICAM-1 and PECAM-1 in BMECs. (A-B) Western blot assay of the expression of CAMs including VCAM-1, ICAM-1 and PECAM-1 in mouse bEnd.3 cells transfected with miR-143-3p minics or miR-nc (n =6 per group, Student' s t-test). (C-D) Detection of the levels of VCAM-1, ICAM-1, and PECAM-1 in hCMEC/D3 cells after the treatment of the proteasome inhibitor MG132 (n =6 per group, Student' s t-test). Data are expressed as mean ± SD. *P < 0.05; **P < 0.01; ***P < 0.001.

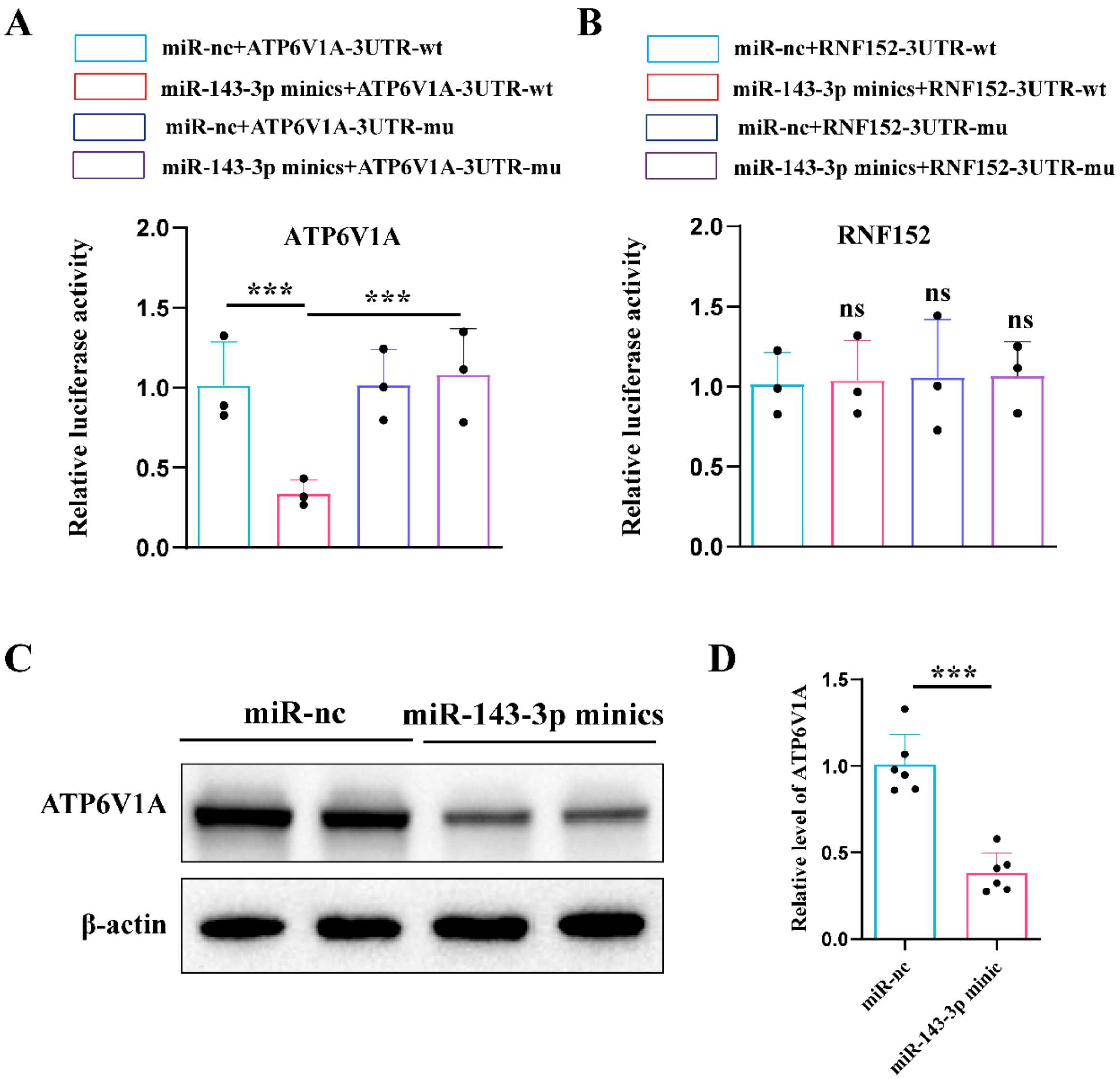

Figure S3. The regulation of miR-143-3p on ATP6V1A. (A) Dual-luciferase analysis in HEK293T cells co-transferred with miR-143-3p mimic and the wild-type or mutated ATP6V1A-3' UTR (n =5 per group, one-way ANOVA). (B) Dual-luciferase analysis in HEK293T cells co-transferred with miR-143-3p mimic and the wild-type or mutated RNF152-3' UTR (n =5 per group, one-way ANOVA). (C-D) Western blot analysis of the protein level of ATP6V1A in hCMEC/D3 cells transferred with the miR-nc or miR-143-3p mimic (n=6 per group, Student' s t-test). Data are presented as means ± SD. $*p < 0.05$,

**p < 0.01, ***p < 0.001.

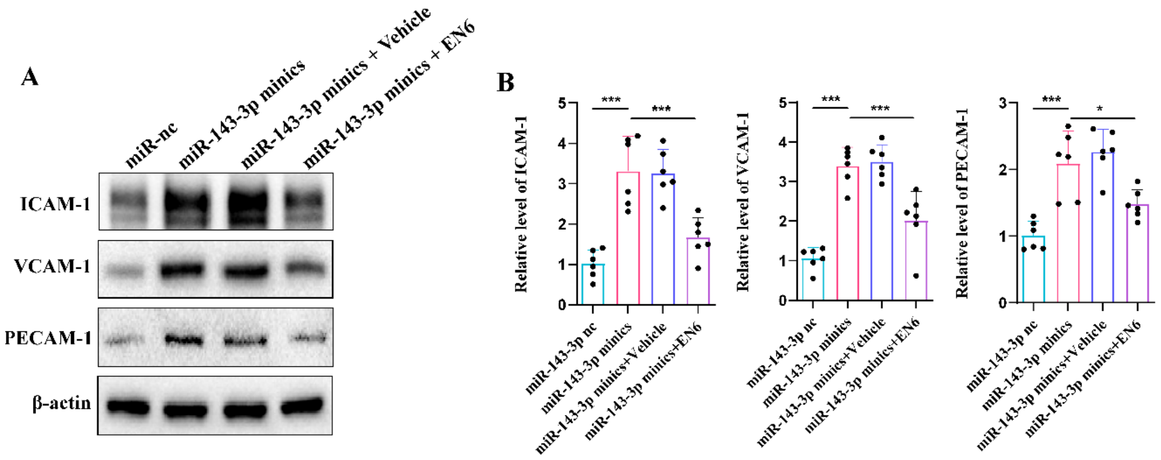

Figure S4. The effects of EN6 treatment on the protein level of VCAM-1, ICAM-1 and PECAM-1 in bEnd.3 cells transferred with miR-nc or miR-143-3p-minics (n =6 per group, one-way ANOVA). Data are presented as means ± SD. *p < 0.05, **p < 0.01, ***p < 0.001.

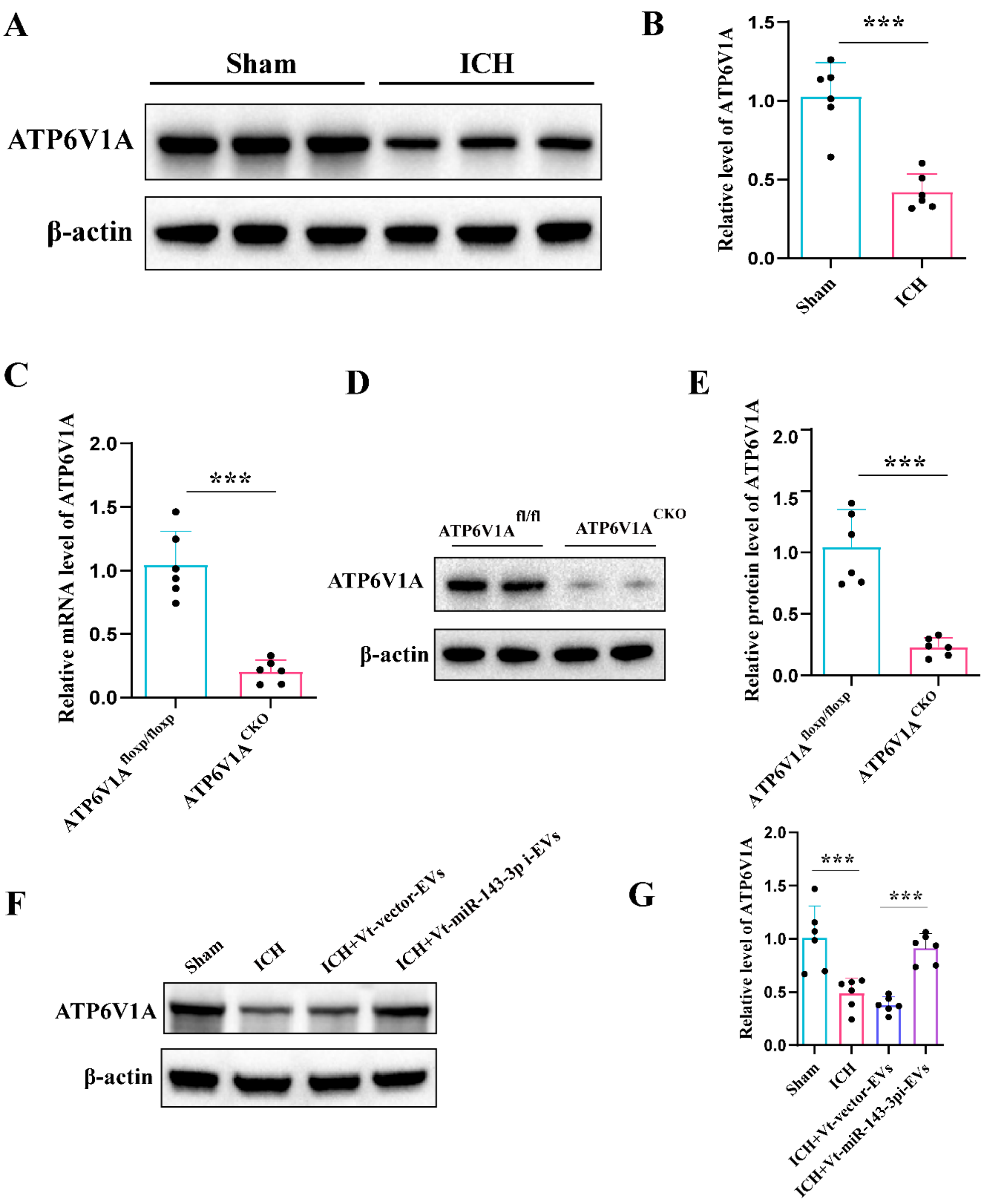

Figure S5. Detection of the level of ATP6V1A in isolated BMECs. (A-B) Western blot assay of the expression of ATP6V1A in isolated BMECs in mice with or without ICH (n =6 per group, Student' s t-test). (C) PCR analysis of the mRNA level of ATP6V1A in isolated BMECs in ATP6V1A^{CKO} mice (n =6 per group, Student' s t-test). (D-E) WB

analysis of the protein level of ATP6V1A in isolated BMECs in ATP6V1A^{CKO} mice (n =6 per group, Student' s t-test). (F-G) Western blot assay of the expression of ATP6V1A in isolated BMECs in ICH mice with or without Vt-miR-143-3pi-EVs treatment (n =6 per group, one-way ANOVA). Data are presented as means ± SD. *p < 0.05, **p < 0.01, ***p < 0.001.

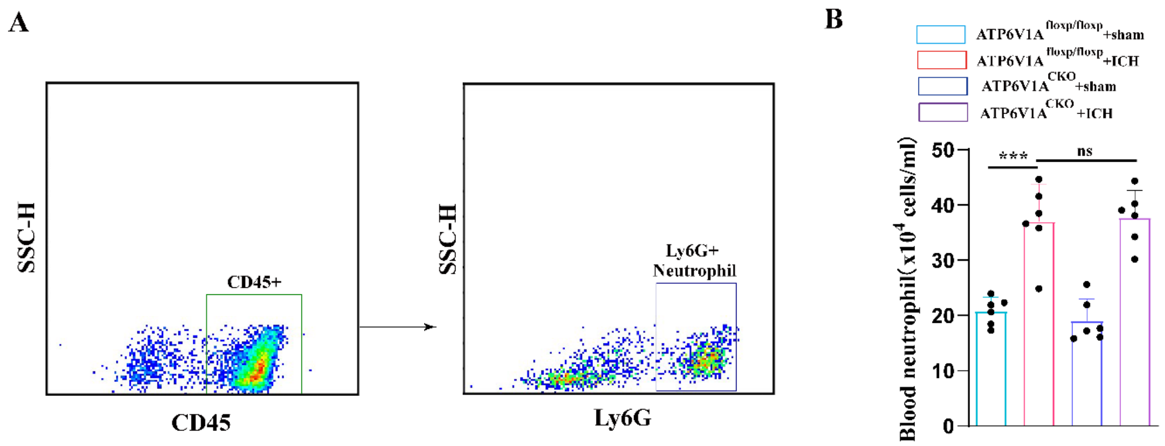

Figure S6. Flow cytometric analysis of the cell numbers of Ly6G+ neutrophils in blood at day 1 post ICH in mice (n =6 per group, one-way ANOVA). Data are presented as means ± SD. *p < 0.05, **p < 0.01, ***p < 0.001.

S-table1. Characteristics of patients with TBI and multivariate logistic regression analysis

Variables	Study population (n=113)	Good outcome (n=67)	Poor outcome (n=46)	Crude OR (95% CI)	Crude P value	Adjusted OR (95% CI)	Adjusted P value
Demographics (age, mean ± SD; male, %)							
Age (y)	48.97(13.36)	47.88(20.41)	50.56(18.46)	0.98-1.02	0.473		
male	76(67.2)	44(65.6)	32(69.5)	0.37-1.87	0.664		
Clinical characteristics							
GCS	12.20 (2.33)	13.23(1.41)	10.69(2.59)	0.43-0.69	<0.001	0.015-0.748	0.024
Tracheotomy n(%)	37(32.74)	2(2.99)	35 (76.09)	21.69-492.93	<0.001	3.82-1.18E+13	0.032
Brain midline shift (mm)	7.39(4.06)	5.63(3.68)	9.96(3.15)	1.22-1.59	<0.05	1.33-64.02	0.024
Laboratory test (mean ± SD)							
WBC (10^9/L)	11.02(5.35)	10.27(4.71)	12.11(6.01)	0.99-1.14	0.075		
AST/ALT	1.43(0.58)	1.28(0.51)	1.65(0.61)	1.53-7.38	0.20		
BG (mmol/L)	8.43(3.51)	7.09(2.58)	10.38(3.8)	1.19-1.65	<0.001	1.30-36.34	0.023
APTT (s)	26.50(7.34)	25.33(6.34)	28.21(8.36)	0.99-1.12	0.58		
FDP (mg/L)	15.47 (20.89)	12.20(20.18)	20.23(21.22)	1.00-1.04	0.58		
miR-143-3p	1.40(0.54)	1.19(0.40)	1.70(0.59)	3.50-29.37	<0.001	4.14-2.52E+6	0.017

Abbreviations: BP, blood pressure; GCS, Glasgow Coma Scale; WBC: white blood cells; AST/ALT, aspartate aminotransferase to alanine aminotransferase ratio; BG blood glucose; APTT, activated partial thromboplastin time; FDP, fibrin degradation products;
